# Whole-genome sequencing of human Pegivirus variant from an Egyptian patient co-infected with hepatitis C virus: a case report

**DOI:** 10.1186/s12985-019-1242-5

**Published:** 2019-11-11

**Authors:** Hany K. Soliman, Mohamed Abouelhoda, Mahmoud N. El Rouby, Ola S. Ahmed, G. Esmat, Zeinab K. Hassan, Mohammed M. Hafez, Dina Ahmed Mehaney, Manojkumar Selvaraju, Rania Kamal Darwish, Yehia A. Osman, Abdel-Rahman N. Zekri

**Affiliations:** 1 Cancer Biology Department, Virology and Immunology Unit, National Cancer Institute, Cairo University, Cairo, 11796 Egypt; 2 Systems and Biomedical Engineering Department, Faculty of Engineering, Cairo University, Cairo, 12613 Egypt; 3 Endemic Medicine and Hepatology Department, Faculty of Medicine, Cairo University, Cairo, 11562 Egypt; 4 Clinical and chemical pathology Department, Faculty of Medicine, Cairo University, Cairo, 11562 Egypt; 5 Integrated gulf biosciences (IGB), Dubai, United Arab Emirates; 6 Botany Department, Faculty of Science, Mansoura University, Mansoura, 33516 Egypt

**Keywords:** Human pegivirus, HCV, Deep NGS, Egyptian patient

## Abstract

**Background:**

Human pegivirus (HPgV) is structurally similar to hepatitis C virus (HCV) and was discovered 20 years ago. Its distribution, natural history and exact rule of this viral group in human hosts remain unclear. Our aim was to determine, by deep next-generation sequencing (NGS), the entire genome sequence of HPgV that was discovered in an Egyptian patient while analyzing HCV sequence from the same patient. We also inspected whether the co-infection of HCV and HPgV will affect the patient response to HCV viral treatment. To the best of our knowledge, this is the first report for a newly isolated HPgV in an Egyptian patient who is co-infected with HCV.

**Case presentation:**

The deep Next Generation Sequencing (NGS) technique was used to detect HCV sequence in hepatitis C patient’s plasma. The results revealed the presence of HPgV with HCV. This co-infection was confirmed using conventional PCR of the HPgV 5′ untranslated region. The patient was then subjected to direct-acting-antiviral treatment (DAA). At the end of the treatment, the patient showed a good response to the HCV treatment (i.e., no HCV-RNA was detected in the plasma), while the HPgV-RNA was still detected. Sequence alignment and phylogenetic analyses demonstrated that the detected HPgV was a novel isolate and was not previously published.

**Conclusion:**

We report a new variant of HPgV in a patient suffering from hepatitis C viral infection.

## Background

There are several techniques that can be used in the detection of the novel virus [1]. In the last decade, the next-generation sequencing (NGS) is a technology perform rapid sequencing [2] and offer a significant way for detecting known viruses, and for discovering novel viruses with several applications in clinical diagnosis [3]. The deep-sequencing is a recently applied technique that yields millions of short sequences and enables investigation of viral diversity and quasi-species. In addition, it enables the simultaneous characterization of different pathogens [4]. Hepatitis C virus (HCV) is an RNA virus that replicates in hepatocytes and liver tissues [5] causing acute, chronic hepatitis, and cirrhosis, which finally may lead to hepatocellular carcinoma [6]. It has been showed that 10–20% of patients infected with chronic HCV are co-infected with human pegivirus (HPgV) in which both HCV and HPgV can be transmitted by parenteral route [7].

The HPgV, which is also known as GB virus C (GBV-C), is a member of the family *Flaviviridae* and the genus Pegivirus [8]. It has a positive-sense RNA genome of ~ 9.3 kb that is translated to produce a single polyprotein [9]. The polyprotein is cleaved by viral protease into smaller viral proteins including two putative envelope proteins (E1 and E2) and several nonstructural proteins (NS2–NS5). The coding region is flanked by long 5′ and 3′ untranslated regions (UTRs) [10]. On the basis of phylogenetic comparisons six genotypes of HPgV have been identified, genotype-1 in West Africa, genotype-2 in North America and Europe, genotype-3 in Asia, genotype-4 in Southeast Asia, genotype-5 in South Africa, and genotype-6 in Indonesia [11, 12]. HPgV is a lymphotropic, non-pathogenic virus which is not associated with any known disease [13]; however its clinical significance still uncertain. It replicates in bone marrow, lymphoid tissue, and peripheral blood mononuclear cells, but is not thought to be hepatotropic [11] while others suggested its hepatotropicity and pathogenicity [14]. Worldwide, the prevalence of HPgV varied from 0.5 to 4% in healthy adults, with much higher levels in particular risk groups, including HIV patients [15]. In Egypt, the high prevalence of HPgV (61%) has been reported in multiple transfused children, and 15% in healthy controls [16]. HPgV viremia can be cleared within the first year of infection followed by protection against reinfection, but it may persist for longer periods [17]. In this case report, we find a new variant for HPgV in a patient suffering from HCV infection by using NGS.

## Case presentation

A 32-year old Egyptian male (bodyweight 80 kg, height 180 cm), infected with HCV, was admitted to Hepatic Viruses Center, Faculty of Medicine, Cairo University, Cairo, Egypt, in April 2018. He was complaining from high levels of aspartate aminotransferase (AST) and alanine aminotransferase (ALT) in the blood (70 and 100 U/L, respectively). A test for serum anti-HCV antibody was positive. At the same time, Quantitative reverse-transcription polymerase chain reaction (qRT-PCR) (using 7500 Fast Real-time PCR system) analysis of RNA from plasma demonstrated that the HCV-RNA level was 2 × 10^6^ IU/ml. The patient didn’t have any history of liver disease, there was no pallor, no jaundice, and no splenomegaly. Also, there were no signs suggesting liver cirrhosis. Laboratory investigations of complete blood picture revealed a hemoglobin value of 14.3 g/dl, a white blood cell count of 5.5x10^3^cells/μl (50% lymphocyte, 5.4% monocyte and 44.6% granules). The platelets count was 2.1 × 10^5^ cells/μl and blood biochemical investigations were normal (Table 1). The biochemical investigations were repeated monthly during the treatment periods. Abdominal ultrasonography identified a fatty liver. Based on these tests, the patient was treated with a combination of DAA for 12 weeks (sofosbuvir 400 mg and daclatasvir 60 mg once a day).

**Table 1:** Clinical features of plasma samples taken throughout the study

Date	AST level (IU/ml) R (up to 41)	ALT level (IU/ml) R (up to 41)	Bilirubin (mg/dl)			Albumin (mg/dl) R(3.5-5.3)	Creatinine (mg/dl) R(0.7-1.4)
			I R (0.1-0.8)	T R (0.3-1.0)	D R (0.1-0.3)		
10th April 2018	70	100	0.9	0.2	0.7	3.9	0.9
13th May 2018	67	54	0.3	0.1	0.2	3.8	0.7
13th June 2018	58	49	0.3	0.1	0.2	3.7	0.8
13th July 2018	58	49	0.3	0.1	0.2	3.7	0.8

*ALT* alanine aminotransferase, *AST* aspartate aminotransferase, *D* direct, *I* indirect, *IU* international units, *T* total, *R* reference range

Before the treatment, the blood sample was collected on EDTA containing tube. RNA sample library was prepared using the TruSeq RNA Sample Preparation Kit v2 (Illumina, San Diego, CA, USA). RNA fragmentation, cDNA synthesis/indexing, PCR amplification/clean-up, and library normalization/pooling steps were conducted according to the manufacturer’s instructions. Sequencing was performed on a MiSeq sequencer with the MiSeq reagent kit v2 (300 cycles; Illumina), as described previously [18]. Paired-end reads (2 × 150 nucleotide) were analyzed to identify the virus. An in-house workflow was used, as previously described [19]. The identification on the HPgV isolate was done as followed: the first step in the pipeline included removal of the adaptor sequence from the reads and trimming low-quality bases using the program Fastx toolkit [http://hannonlab.cshl.edu/fastx_toolkit]. The next step was to map the reads to a database of viral sequences using the specific programs SNAP DB [https://www.freewarefiles.com/Snap-DB_program_86429.html] and BWA [http://bio-bwa.sourceforge.net]. The reads mapped to the same taxa were grouped together and assembled with SPAdes program, to yield a set of contigs. The Un-mapped reads (which could represent novel sub-sequences) were collected and assembled with the contigs to close any potential gaps and to improve the assembly. Then final contigs were aligned to the reference genome of each group using pairwise clustalw2. Furthermore, the whole viral genome sequences for the most significant group were collected from the GenBank database and the contig was aligned to each of them in order to check if the target sequence has better similarity to a sequence rather than those in the RefSeq database. The assembly yielded a single contig of 9370 bases for the HPgV sequence and another contig of 9291 bases for the HCV sequence, which was submitted to GenBank database and assigned with accession numbers MK234885 and MK799639, respectively. The contig was identified as HPgV by matching the sequences to the set of whole-genome pegivirus sequences in the GenBank database. The most significant hits were 75 sequences with a minimum average depth 100× and minimum 90% genome coverage (Additional file 1). The supplementary file also included the statistics for our sequence alignment and each pegivirus sequence in the hit list. These results indicate a high level of confidence in viral identification and give a strong hint regarding its genotype.

**Fig. 1:**
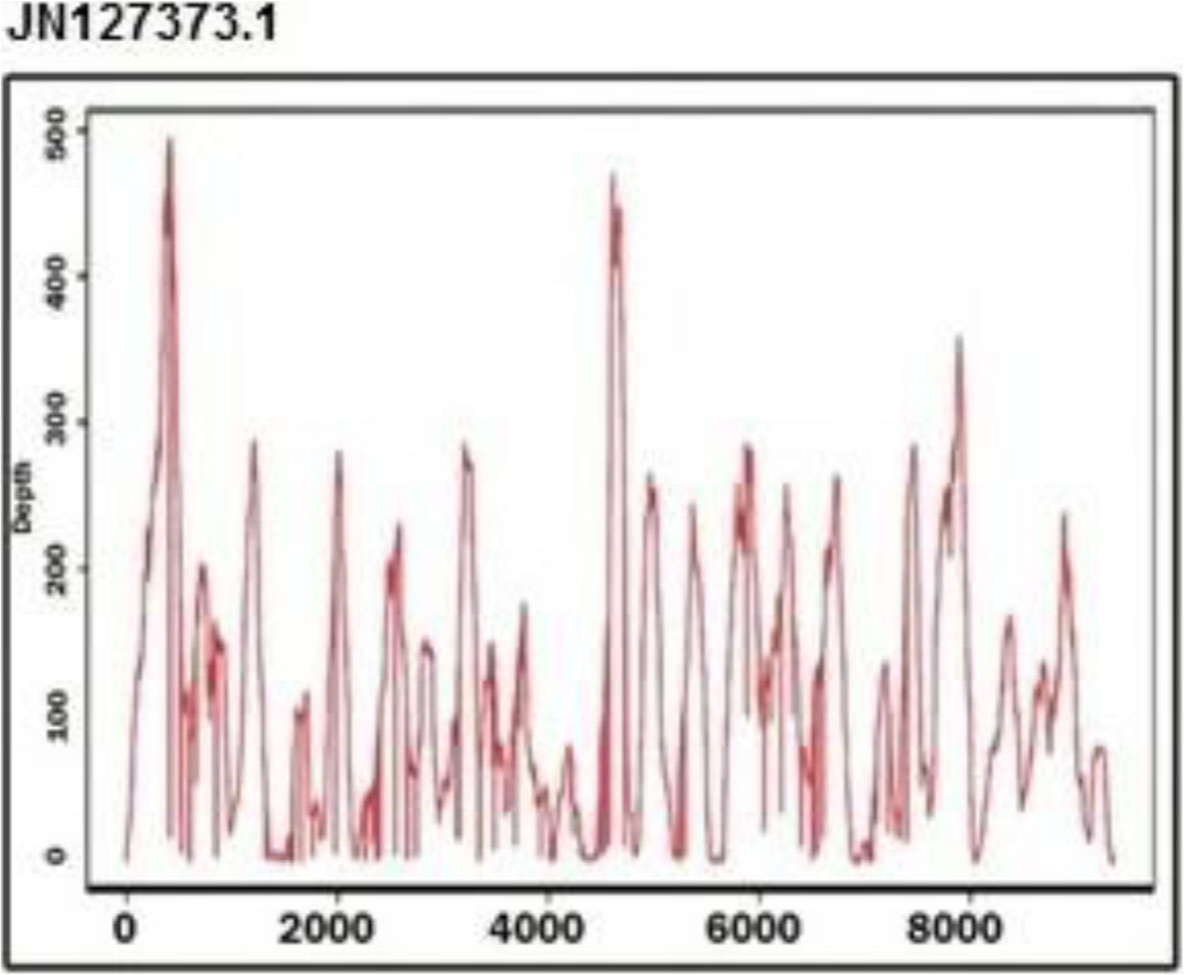
Coverage plot for the Egyptian HPgV isolate sequence against the best matched genome JN127373. The X-axis is the genome coordinate (in bps) and the Y axis is the number of NGS reads covering that position (depth). The plot was generated using python script from the pileup file generated from the alignment SAM File

**Fig. 2:**
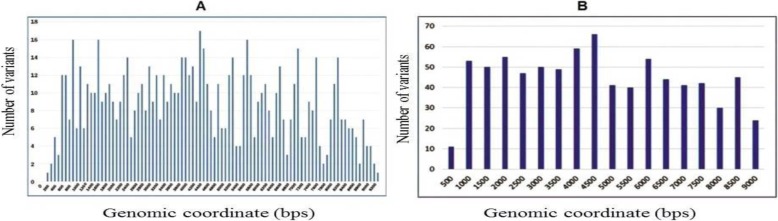
Distribution of variants throughout the genome of a novel human pegivirus isolate. **a** The chart shows the number of variants within windows of size 100 bps sliding over the genomic sequence. **b** The chart shows the number of variants within windows of size 500 bps

The best alignment in this list was to the sequence JN127373.1 (GB virus C isolate UU1), which had 98.77% coverage, 91.1% identity, and 172× average depth (Fig. 1) (Additional file 2). The variants were distributed throughout the whole genome (Fig. 2). Further data analysis using DNAStar software of 9370 bp for HPgv genotype-2 showed the identification of 808 SNPs that consisted of 806 coding SNPs (cSNPs) and 2 non-coding SNPs (Additional file 3). According to the type of SNPs for HPgV genome, transition substitutions were more predominant than transversions (73.2% vs 26.8%). Transitions C↔T and A↔G are over-represented with 46 and 26.3% of the total substitutions respectively. The frequency of transitions between coding region were significantly different (73.2% vs 26.8% respectively; χ2 = 5.86, *P < 0.01*). This confirms that SNPs occur more frequently as transitions in coding regions than in non-coding regions.

**Fig. 3:**
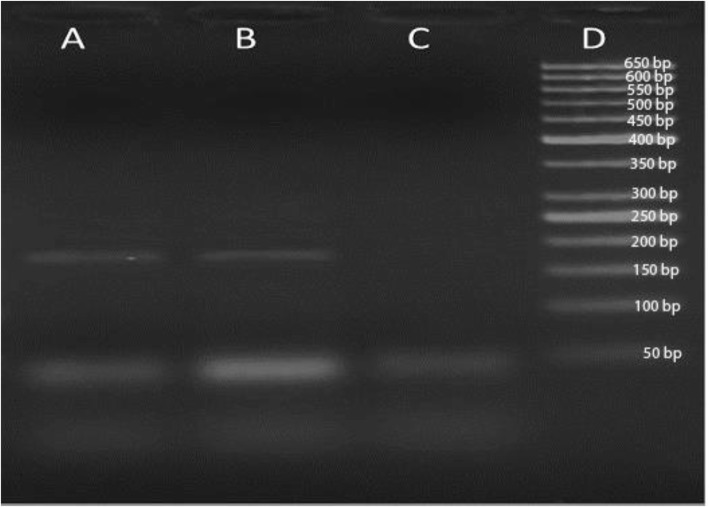
Identification of human pegivirus (HPgV) infection by Revese transcriptase polymerase chain reaction (RT-PCR). Plasma samples before (lane **a**) and after (lane **b**) viral treatment with daclatasvir and sofosbuvir. Viral RNA was extracted using viral RNA extraction kit followed by synthesis the complementary DNA (cDNA) with a high-capacity cDNA kit (Applied Biosystems). The PCR reaction targeting the 5′ UTR was performed in the thermal cycle, for 40 cycles to amplify a 173 bp fragment. PCR product was loaded on 2% gel and electrophoresed for 20 min at 120 V and 90 A after that the gel was visualized and imaging using photo-documentation system (Biometria). Lane **c** is a negative control, and lane **d** contains a DNA marker (50 bp ladder)

After HCV-treatment, HCV-RNA was no longer detected in the plasma by qRT-PCR, whereas HPgV-RNA was still detected with conventional RT-PCR (Fig. 3). Conventional RT-PCR was done as followed: the complementary DNA (cDNA) synthesis was performed with a high-capacity cDNA kit (Applied Biosystems) according to the manufacturer’s instructions. The PCR reaction targeting the 5′ UTR consisted of 1 × PCR buffer, 200 μM dNTPs, 1.5 mM MgCl_2_, 300 nM UTR-F (5′-GATGCCAGGGTTGGTAGGTC-3′; positions 120–139), 300 nM UTR-R (5′-CTCGGTTTAACGACGAGCCT-3′; positions 293–274), 2.5 U Hot start *Taq* DNA polymerase (Qiagen, Germany), and 1 μl cDNA. Thermal-cycling parameters were as follows: an initial denaturation for 5 min at 95 °C, followed by 40 cycles of denaturation for 60 s at 95 °C, annealing for 90 s at 55 °C, and extension for 120 s at 72 °C; final extension for 10 min at 72 °C. The PCR product was subjected to electrophoresis on 2.5% agarose gel, stained with ethidium bromide (0.5 μg/ml) and visualized with ultraviolet trans-illuminator.

**Fig. 4:**
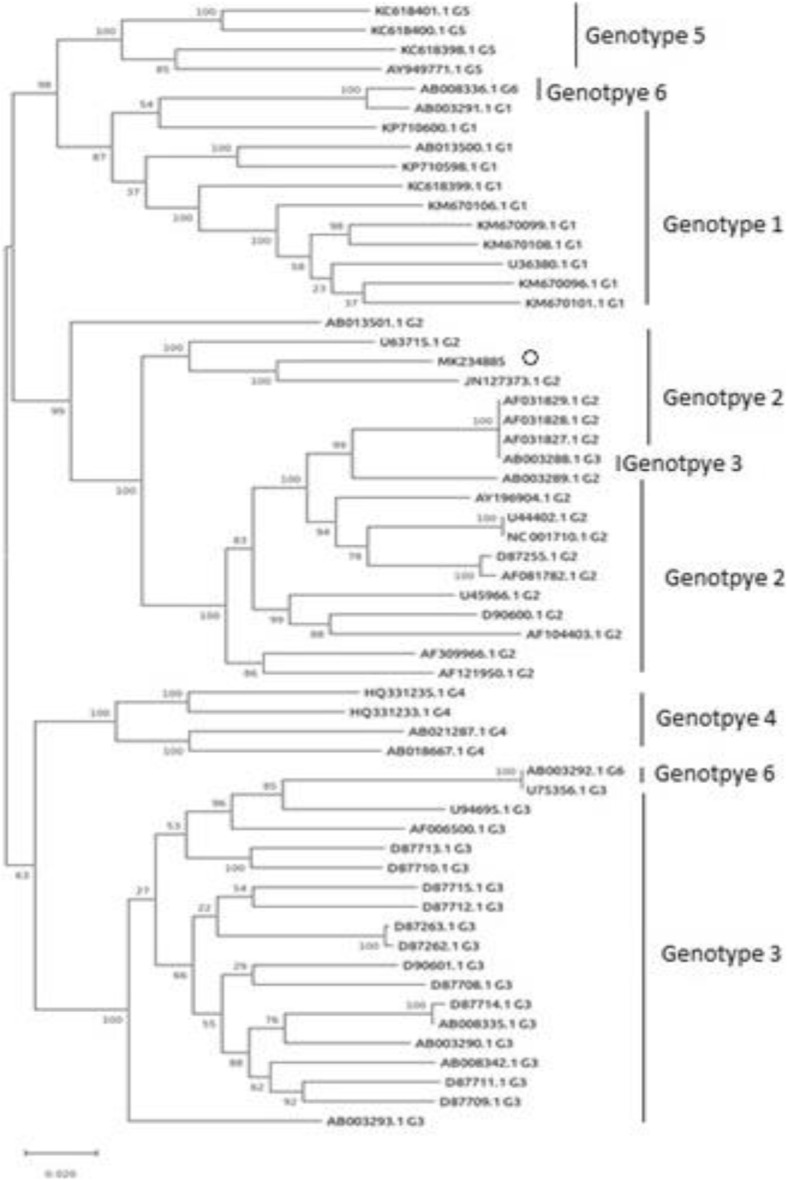
Phylogenetic relationship between whole-genome sequences of a human pegivirus (HPgV) isolate (MK234885) symbolled by (0) and different HPgV representative isolates. This tree produced from an alignment of 58 full coding genomes including our new isolate full coding sequence described herein. Phylogenetic analysis was undertaken using MEGA X software and constructed using Maximum Likelihood method with default parameters (including bootstrapping with 1000 replicates). Comparison with six known genotypes of HPgV, which is also known as GB virus C or hepatitis G virus, identified the novel isolate as a previously unidentified variant of genotype 2

To confirm the HPgV genotyping of the Egyptian isolate, the genotype information was extracted for most of the sequences from the literature and inferred the others using BLAST analysis (Additional file 1). Then multiple sequence alignment was performed using DNADynamo software, followed by phylogenetic inference using our isolate sequence and the list of all whole-genome sequences of pegivirus in the hit list. For phylogenetic tree reconstruction, the analysis was conducted with MEGA X software [20] using Maximum Likelihood method with default parameters (including bootstrapping with 1000 replicates) and confirmed that this isolate resembles HPgV genotype-2 (Fig. 4). Also, based on the bioinformatics analysis of reads obtained by deep sequencing of a relevant genome sequence the genotype of HCV was 4n. Alignments of the NS5A and NS5B protein structures of HCV and HPgV revealed that there were many differences in the protein sequences between HPgV and HCV (Additional files 4 and 5).

## Discussion and conclusions

In spite of the discovery of the first pegiviruses for 20 years, the natural history of this viral group and its pathogenicity remains unclear. The pathogenicity of HPgV has caused many controversies. While some studies documented that HPgV is a lymphotropic virus and its infection considered nonpathogenic [14, 21, 22], others showed that it is a hepatotropic virus and pathogenic to humans and animals [14]. This contradiction may be due to the occurrence of mixed infection between HPgV and other hepatitis viruses in which patients with single infection are rare [14]. The advanced molecular studies (the sequence-specific, sequence-independent PCR-based approaches, and massive deep sequencing approaches) improved the understanding of genetic diversity of viruses. By using these approaches, we were able to sequence the new variant of HPgV that was present in the Egyptian HCV-infected patient. According to the bioinformatics analysis, we confirmed the presence of a new variant of HPgV genotype-2. The HPgV genotype-2 had been identified in North and South America, Europe, East Africa, and the Indian subcontinent [13].

At the end of the treatment, the patient responded to the DAA treatment in spite of being infected with HPgV. In the present case, the co-infection of HPgV with HCV didn’t result in a significant change in the clinical presentation, biochemical changes, viral load or response to treatment [23]. Daclatasvir and sofosbuvir are being used as inhibitors of the NS5A and NS5B proteins, of HCV respectively [24], since both of them have key roles in the replication of HCV RNA [25]. The differences in treatment response between HPgV and HCV infected patient may be due to the different structures of NS5A and NS5B in the two viruses. To the best of our knowledge, this is the first study from Egypt and the Middle East to analyze the whole genome sequence of HPgV using deep sequencing technique.

In conclusion, this is the first study from Egypt and the Middle East reporting the presence of a new variant of HPgV genotype-2 in Egyptian patient infected with HCV. The whole-genome sequencing identified this isolate as a novel variant of HPgV, and provided evidence that co-infection by HPgV and HCV has no influence on patients’ response to HCV treatment.

## Supplementary information

**Additional file 1:** The most significant hits of HPgV sequence.

**Additional file 2:** The best alignment of our sequence with ref. seq. JN127373.1.

**Additional file 3:** Identification of 808 SNPs.

**Additional file 4:** Alignments of the NS5A protein structure of HCV and HPgV.

**Additional file 5:** Alignments of the NS5B protein structure of HCV and HPgV.

## Data Availability

All data and material are available in this manuscript and in additional files.

## Abbreviations

ALT: Alanine aminotransferase
AST: Aspartate aminotransferase
BLAST: Basic Local Alignment Search Tool
cDNA: complementary DNA
DAA: Direct-acting-antiviral
DNA: Deoxyribonucleic acid
EDTA: Ethylenediamine tetraacetic acid
GBV-C: GB virus C
HCV: Hepatitis C virus
HPgV: Human pegivirus
NGS: Next generation sequencing
NS5A: Non-structural protein 5A
NS5B: Non-structural protein 5B
PCR: Polymerase chain reaction
qRT-PCR: quantitative reverse-transcription PCR
RNA: Ribonucleic acid
SNPs: Single nucleotide polymorphisms
UTRs: Untranslated regions
