# Supplementary material for: Whole-genome sequencing of human Pegivirus variant from an Egyptian patient co-infected with hepatitis C virus: a case report
Source: Virol J. 2019 Nov 11;16:132. doi: 10.1186/s12985-019-1242-5 (PMC6849219; doi:10.1186/s12985-019-1242-5)
Supplement: Supplementary file 2 — Additional file 2: The best alignment of our sequence with ref. seq. JN127373.1. [file 12985_2019_1242_MOESM2_ESM.pdf]

```
JN127373.1      CACTGGGTGCAAGCCCCAGAAACCGACGCCTATCTAAGTAGACGCAATGACT 52
Our sequence    CACTGGGTGCAAGCCCCAGAAACCGACGCCTATCTAAGTAGACGCAATGACT 52
Score           ****************************************************

JN127373.1      CGGCGCCGACCCGGCGACCGGCCAAAAGGTGGTGGATGGGTGATGCCAGGGT 104
Our sequence    CGGCGCCGACCCGGCGACCGGCCAAAAGGTGGTGGATGGGTGATGCCAGGGT 104
Score           ****************************************************

JN127373.1      TGGTAGGTCGTAAATCCCGGTCATCTTGGTAGCCACTATAGGTGGGTCTTAA 156
Our sequence    TGGTAGGTCGTAAATCCCGGTCATCTTGGTAGCCACTATAGGTGGGTCTTAA 156
Score           ****************************************************

JN127373.1      GGGAAGGTTAAGATTCCTCTTGTGCCTGTGGCGAGACAGCGCACGGTCCACA 208
Our sequence    GGGAAGGTTAAGATTCCTCTTGTGCCTGTGGCGAGACAGCGCACGGTCCACA 208
Score           ****************************************************

JN127373.1      GGTGTTGGCCCTACCGGTGTGAATAAGGGCCCGACGTCAGGCTCGTCGTTAA 260
Our sequence    GGTGTTGGCCCTACCGGTGTGAATAAGGGCCCGACGTCAGGCTCGTCGTTAA 260
Score           ****************************************************

JN127373.1      ACCGAGACCGACACCCACCCGGGCAAACGACGCTCACGTACGGTCCACGTCG 312
Our sequence    ACCGAGACCGACACCCACCCGGGCAAACGACGCTCACGTACGGTCCACGTCG 312
Score           ****************************************************

JN127373.1      CCCTTCAATGTCTCTCTTGACCAATAGGCTTTGCCGGCGAGTTGACAAGGAC 364
Our sequence    CCCTTCAATGTCTCTCTTGACCAATAGGCTTTGCCGGCGAGTTGACAAGGAC 364
Score           ****************************************************
```

```
JN127373.1        CAGTGAGGGCCGGGGGCGAGGGGGAAGGACCCCCTCGCCCTGCCCATTCCGG 416
Our sequence      CAGTGAGGGCCGGGGGCGAGGGGGAAGGACCCCCTCGCCCTGCCCATTCCGG 416
Score             ****************************************************

JN127373.1        TGGGACGGGAAATGCATGGGGCCACCCAGCTCCGCGGCGGCCTGCAGCCGGG 468
Our sequence      TGGGACGGGAAATGCATGGGGCCACCCAGCTCCGCGGCGGCCTGCAGCCGGG 468
Score             ****************************************************

JN127373.1        GTAGCCCAAGAGCCTTCGGGTGAGGGCGGGTGGCATTTTTCTTTCTTAACCG 520
Our sequence      GTAGCCCAAGAGCCTTCGGGTGAGGGCGGGTGGCATTTTTCTTTCTTAACCG 520
Score             ****************************************************

JN127373.1        ATCATGGCAGTCCTTCTGCTCTTCTTCGTGGTTGAGGCCGGGGCCATTCTGG 572
Our sequence      ATCATGGCAGTCCTTCTGCTCTTCTTCGTGGTTGAGGCCGGGGCCATTCTGG 572
Score             ****************************************************

JN127373.1        CCCCGGCCACCCACGCTTGTCGGGCTGACGGGCAATATTTCCTCACGAACTG 624
Our sequence      CCCCGGCCACCCACGCTTGTCGGGCTGACGGGCAATATTTCCTCACGAACTG 624
Score             ****************************************************

JN127373.1        TTGTGCCCTTGAGGACATAGGGTTCTGCCTGGAAGGTGGATGCCTTGTGGCC 676
Our sequence      TTGTGCCCTTGAGGACATAGGGTTCTGCCTGGAAGGTGGATGCCTTGTGGCC 676
Score             ****************************************************

JN127373.1        CTGGGGTGTACGGTTTGTACTGACAAATGCTGGCCACTGTATCAGGCGGGTT 728
Our sequence      CTGGGGTGTACGGTTTGTACTGACAAATGCTGGCCACTGTATCAGGCGGGTT 728
Score             ****************************************************
```

```
JN127373.1        TGGCTGTGCGGCCTGGCAAGTCCGCAGCCCAGCTCGTGGGGGAGCTTGGGAG 780
Our sequence      TGGCTGTGCGGCCTGGCAAGTCCGCAGCCCAGCTCGTGGGGGAGCTTGGGAG 780
Score             ****************************************************

JN127373.1        CCTTTTCGGGCCCCTATCGGTGTCCGCCTACGTGGCGGGTATCTTGGGGCTC 832
Our sequence      CCTTTTCGGGCCCCTATCGGTGTCCGCCTACGTGGCGGGTATCTTGGGGCTC 832
Score             ****************************************************

JN127373.1        GGGGAGGTTTACTCGGGAGTCCTCACGGTTGGGGTCGCGTTGACGCGTCGGG 884
Our sequence      GGGGAGGTTTACTCGGGAGTCCTCACGGTTGGGGTCGCGTTGACGCGTCGGG 884
Score             ****************************************************

JN127373.1        TCTACCCGGTCCCGAACCTAACGTGTGCAGTAGAGTGTGAGTTAAAGTGGGA 936
Our sequence      TCTACCCGGTCCCGAACCTAACGTGTGCAGTAGAGTGTGAGTTAAAGTGGGA 936
Score             ****************************************************

JN127373.1        GAGTGAGTTTTGGAGATGGACTGAGCAGTTGGCCTCCAATTACTGGATTCTG 988
Our sequence      GAGTGAGTTTTGGAGATGGACTGAGCAGTTGGCCTCCAATTACTGGATTCTG 988
Score             ****************************************************

JN127373.1        GAATATCTCTGGAAGGTACCATTTGACTTTTGGCGGGGAGTGATGAGCCTGA 1040
Our sequence      GAATATCTCTGGAAGGTACCATTTGACTTTTGGCGGGGAGTGATGAGCCTGA 1040
Score             ****************************************************

JN127373.1        CACCCCTGTTGGTGTGCGTGGCGGCCCTACTCCTGCTAGAGCAAAGGCTTGT 1092
Our sequence      CACCCCTGTTGGTGTGCGTGGCGGCCCTACTCCTGCTAGAGCAAAGGCTTGT 1092
Score             ****************************************************
```

```
JN127373.1      TATGGTGTTCCTCCTGGTGACCATGGCTGGCATGTCGCAAGGCGCGCCCGCT 1144
Our sequence    TATGGTGTTCCTCCTGGTGACCATGGCTGGCATGTCGCAAGGCGCGCCCGCT 1144
Score           ****************************************************

JN127373.1      TCGGTCCTTGGATCACGCCCATTCGATCGTGGGCTGACCTGGCAGGCCTGCT 1196
Our sequence    TCGGTCCTTGGATCACGCCCATTCGATCGTGGGCTGACCTGGCAGGCCTGCT 1196
Score           ****************************************************

JN127373.1      CGTGCAGGGCAAACGGCTCGCGCGTGCCGACTGGGGAGAAGGTTTGGGAACG 1248
Our sequence    CGTGCAGGGCAAACGGCTCGCGCGTGCCGACTGGGGAGAAGGTTTGGGAACG 1248
Score           ****************************************************

JN127373.1      TGGGAACGTCACACTGTTGTGTGACTGCCCCAACGGCCCCTGGGTATGGTTG 1300
Our sequence    TGGGAACGTCACACTGTTGTGTGACTGCCCCAACGGCCCCTGGGTATGGTTG 1300
Score           ****************************************************

JN127373.1      CCAGCCGTTTGCCAAGCAGTCGGCTGGGGCGACCCTATCACTCATTGGAGCC 1352
Our sequence    CCAGCCGTTTGCCAAGCAGTCGGCTGGGGCGACCCTATCACTCATTGGAGCC 1352
Score           ****************************************************

JN127373.1      ACGGACAAAATCAGTGGCCCCTCTCGTGTCCCCAATATGTTTATGGTGCCGT 1404
Our sequence    ACGGACAAAATCAGTGGCCCCTCTCGTGTCCCCAATATGTTTATGGTGCCGT 1404
Score           ****************************************************

JN127373.1      TTCAGTGACCTGCGTGTGGGGTTCTGTCTCTTGGTTTGCCTCCACTGGCGGG 1456
Our sequence    TTCAGTGACCTGCGTGTGGGGTTCTGTCTCTTGGTTTGCCTCCACTGGCGGG 1456
Score           ****************************************************
```

```
JN127373.1      CGCGACTCGAGGATCGATGTGTGGAACTTGGTGCCGGTTGGATCAGCCAGCT 1508
Our sequence    CGCGACTCGAGGATCGATGTGTGGAACTTGGTGCCGGTTGGATCAGCCAGCT 1508
Score           ****************************************************
```

```
JN127373.1      GCACCATAGCCGCTCTCGGTTCTTCGGATCGCGATGTCGTAGTGGAGCTCTC 1560
Our sequence    GCACCATAGCCGCTCTCGGTTCTTCGGATCGCGATGTCGTAGTGGAGCTCTC 1560
Score           ****************************************************
```

```
JN127373.1      TGAGTGGGGGATACCCTGCGTGACATGCATTCTGGATCGTCGGCCGGCCTCG 1612
Our sequence    TGAGTGGGGGATACCCTGCGTGACATGCATTCTGGATCGTCGGCCGGCCTCG 1612
Score           ****************************************************
```

```
JN127373.1      TGCGGCACCTGTGTGAGGGACTGCTGGCCCGAAACCGGGTCAGTTAGATTCC 1664
Our sequence    TGCGGCACCTGTGTGAGGGACTGCTGGCCCGAAACCGGGTCAGTTAGATTCC 1664
Score           ****************************************************
```

```
JN127373.1      CTTTCCATCGGTGTGGCGCGGGCCCCCGGCTGACTAAGGACTTGGAAGCCGT 1716
Our sequence    CTTTCCATCGGTGTGGCGCGGGCCCCCGGCTGACTAAGGACTTGGAAGCCGT 1716
Score           ****************************************************
```

```
JN127373.1      ACCCTTCGTCAACAGGACAACTCCCTTCACCATTAGAGGGCCCCTGGGAAAC 1768
Our sequence    ACCCTTCGTCAACAGGACAACTCCCTTCACCATTAGAGGGCCCCTGGGAAAC 1768
Score           ****************************************************
```

```
JN127373.1      CAGGGGAGAGGCAATCCGGTGCGGTCGCCCCTGGGTTTTGGGTCCTACACCA 1820
Our sequence    CAGGGGAGAGGCAATCCGGTGCGGTCGCCCCTGGGTTTTGGGTCCTACACCA 1820
Score           ****************************************************
```

```
JN127373.1        TGACCAAGATCCGGGACTCTCTACATTTGGTGAAATGTCCCACACCAGCTAT 1872
Our sequence      TGACCAAGATCCGGGACTCTCTACATTTGGTGAAATGTCCCACACCAGCTAT 1872
Score             ****************************************************

JN127373.1        AGAGCATCCCACTGGGACGTTCGGGTTCTTCCCTGGACAGCCGCCCCTCAAC 1924
Our sequence      AGAGCATCCCACTGGGACGTTCGGGTTCTTCCCTGGACAGCCGCCCCTCAAC 1924
Score             ****************************************************

JN127373.1        AACTGCATGCTTCTTGGCACTGAGGTGTCTGAGGCATTAGGTGGGGCTGGCT 1976
Our sequence      AACTGCATGCTTCTTGGCACTGAGGTGTCTGAGGCATTAGGTGGGGCTGGCT 1976
Score             ****************************************************

JN127373.1        TGACGGGGGGGTTCTACGAGCCCCTGGTGCGCAGGTGTTCGGAGCTGATGGG 2028
Our sequence      TGACGGGGGGGTTCTACGAGCCCCTGGTGCGCAGGTGTTCGGAGCTGATGGG 2028
Score             ****************************************************

JN127373.1        CCGCCGCAATCCAGTTTGTCCGGGGTTTGCATGGCTCTCCTCGGGCAGGCCT 2080
Our sequence      CCGCCGCAATCCAGTTTGTCCGGGGTTTGCATGGCTCTCCTCGGGCAGGCCT 2080
Score             ****************************************************

JN127373.1        GATGGGTTTATACATGTTCAGGGGCACTTGCAGGAGGTGGATGCGGGCAACT 2132
Our sequence      GATGGGTTTATACATGTTCAGGGGCACTTGCAGGAGGTGGATGCGGGCAACT 2132
Score             ****************************************************

JN127373.1        TCATTCCGCCCCCACGCTGGCTGCTCCTGGATTTTGTATTTGTCCTGTTATA 2184
Our sequence      TCATTCCGCCCCCACGCTGGCTGCTCCTGGATTTTGTATTTGTCCTGTTATA 2184
Score             ****************************************************
```

```
JN127373.1      CCTGATGAAGCTGGCTGAGGCTAGGTTGGTTCCGCTCATACTTCTCCTGCTA 2236
Our sequence    CCTGATGAAGCTGGCTGAGGCTAGGTTGGTTCCGCTCATACTTCTCCTGCTA 2236
Score           ****************************************************

JN127373.1      TGGTGGTGGGTGAACCAGTTGGCGGTCCTCGGACTGCCGGCTGCGCACGCCG 2288
Our sequence    TGGTGGTGGGTGAACCAGTTGGCGGTCCTCGGACTGCCGGCTGCGCACGCCG 2288
Score           ****************************************************

JN127373.1      CCGTGGCGGGGGAGGTGTTTGCGGGGCCAGCCCTGTCATGGTGTCTGGGCCT 2340
Our sequence    CCGTGGCGGGGGAGGTGTTTGCGGGGCCAGCCCTGTCATGGTGTCTGGGCCT 2340
Score           ****************************************************

JN127373.1      CCCCTTCGTGAGCATGATCCTGGGTCTAGCAAACCTGGTGTTGTACTTCCGT 2392
Our sequence    CCCCTTCGTGAGCATGATCCTGGGTCTAGCAAACCTGGTGTTGTACTTCCGT 2392
Score           ****************************************************

JN127373.1      TGGATGGGGCCCCAGCGCCTGATGTTTCTCGTGTTGTGGAAGCTCGCTCGGG 2444
Our sequence    TGGATGGGGCCCCAGCGCCTGATGTTTCTCGTGTTGTGGAAGCTCGCTCGGG 2444
Score           ****************************************************

JN127373.1      GAGCCTTCCCGCTGGCACTTCTGATGGGGATCTCGGCGACCCGCGGCCGCAC 2496
Our sequence    GAGCCTTCCCGCTGGCACTTCTGATGGGGATCTCGGCGACCCGCGGCCGCAC 2496
Score           ****************************************************

JN127373.1      CTCAGTGCTCGGCGCCGAATTCTGCTTCGATGTCACATTCGAGGTGGACACT 2548
Our sequence    CTCAGTGCTCGGCGCCGAATTCTGCTTCGATGTCACATTCGAGGTGGACACT 2548
Score           ****************************************************
```

```
JN127373.1      TCGGTTCTGGGATGGGTGGTGGCCAGCGTGGTAGCTTGGGCCATAGCGCTTC 2600
Our sequence    TCGGTTCTGGGATGGGTGGTGGCCAGCGTGGTAGCTTGGGCCATAGCGCTTC 2600
Score           ****************************************************

JN127373.1      TGAGCTCAATGAGTGCAGGAGGGTGGAAGCACAAAGCTGTGATTTACAGGAC 2652
Our sequence    TGAGCTCAATGAGTGCAGGAGGGTGGAAGCACAAAGCTGTGATTTACAGGAC 2652
Score           ****************************************************

JN127373.1      GTGGTGCAAGGGGTATCAGGCGGTACGTCAACGGGTGGTTCGCAGCCCCCTC 2704
Our sequence    GTGGTGCAAGGGGTATCAGGCGGTACGTCAACGGGTGGTTCGCAGCCCCCTC 2704
Score           ****************************************************

JN127373.1      GGGGAGGGGCGGCCTACTAAGCCTTTGACTTTCGCTTGGTGCTTGGCCTCGT 2756
Our sequence    GGGGAGGGGCGGCCTACTAAGCCTTTGACTTTCGCTTGGTGCTTGGCCTCGT 2756
Score           ****************************************************

JN127373.1      ACATCTGGCCTGACGCGGTGATGATGGTGGTGGTGGCTATGGTGCTTCTCTT 2808
Our sequence    ACATCTGGCCTGACGCGGTGATGATGGTGGTGGTGGCTATGGTGCTTCTCTT 2808
Score           ****************************************************

JN127373.1      CGGCTTGTTCGATGCGTTGGACTGGGCCTTAGAGGAGCTCCTGGTCTCTCGG 2860
Our sequence    CGGCTTGTTCGATGCGTTGGACTGGGCCTTAGAGGAGCTCCTGGTCTCTCGG 2860
Score           ****************************************************

JN127373.1      CCCTCGTTGCGCCGATTGGCTCGGGTGGTTGAGTGTTGTGTGACGGCTGGTG 2912
Our sequence    CCCTCGTTGCGCCGATTGGCTCGGGTGGTTGAGTGTTGTGTGACGGCTGGTG 2912
Score           ****************************************************
```

```
JN127373.1      AAAGGGCCACTACTGTGAGGTTGGTCTCCAAGATGTGCGCGAGGGGGGTCTA 2964
Our sequence    AAAGGGCCACTACTGTGAGGTTGGTCTCCAAGATGTGCGCGAGGGGGGTCTA 2964
Score           ****************************************************

JN127373.1      TTTGTTCGACCATATGGGCTCCTTCTCGCGCTCTGTCAAGGAGCGCTTGCTG 3016
Our sequence    TTTGTTCGACCATATGGGCTCCTTCTCGCGCTCTGTCAAGGAGCGCTTGCTG 3016
Score           ****************************************************

JN127373.1      GAATGGGACGCGGCTCTTGAACCCCTGTCATTCACCAGGACGGACTGTCGCA 3068
Our sequence    GAATGGGACGCGGCTCTTGAACCCCTGTCATTCACCAGGACGGACTGTCGCA 3068
Score           ****************************************************

JN127373.1      TCATAAGAGATGCCGCCAGGACCCTCTCGTGCGGCCAGTGTGTCATGGGCTT 3120
Our sequence    TCATAAGAGATGCCGCCAGGACCCTCTCGTGCGGCCAGTGTGTCATGGGCTT 3120
Score           ****************************************************

JN127373.1      GCCCGTGGTAGCACGTCGCGGCGATGAGGTGCTGATAGGTGTCTTCCAAGAT 3172
Our sequence    GCCCGTGGTAGCACGTCGCGGCGATGAGGTGCTGATAGGTGTCTTCCAAGAT 3172
Score           ****************************************************

JN127373.1      GTGAACCATTTGCCTCCCGGGTTTGTCCCAACCGCGCCAGTTGTCATCCGGC 3224
Our sequence    GTGAACCATTTGCCTCCCGGGTTTGTCCCAACCGCGCCAGTTGTCATCCGGC 3224
Score           ****************************************************

JN127373.1      GGTGCGGGAAGGGCTTTCTCGGAGTCACAAAGGCAGCCTTGACGGGTCGGGA 3276
Our sequence    GGTGCGGGAAGGGCTTTCTCGGAGTCACAAAGGCAGCCTTGACGGGTCGGGA 3276
Score           ****************************************************
```

```
JN127373.1      CCCTGATCTACACCCAGGGAACGTCATGGTGTTGGGGACGGCTACCTCGCGC 3328
Our sequence    CCCTGATCTACACCCAGGGAACGTCATGGTGTTGGGGACGGCTACCTCGCGC 3328
Score           ****************************************************

JN127373.1      AGCATGGGCACATGTCTGAACGGGTTGCTGTTCACTACTTTCCATGGGGCTT 3380
Our sequence    AGCATGGGCACATGTCTGAACGGGTTGCTGTTCACTACTTTCCATGGGGCTT 3380
Score           ****************************************************

JN127373.1      CTTCCCGAACCATCGCCACGCCCGTGGGGGCCCTTAATCCCAGGTGGTGGTC 3432
Our sequence    CTTCCCGAACCATCGCCACGCCCGTGGGGGCCCTTAATCCCAGGTGGTGGTC 3432
Score           ****************************************************

JN127373.1      GGCCAGTGATGATGTGACCGTGTACCCTCTCCCAGACGGGGCAACCTCGTTA 3484
Our sequence    GGCCAGTGATGATGTGACCGTGTACCCTCTCCCAGACGGGGCAACCTCGTTA 3484
Score           ****************************************************

JN127373.1      GCGCCTTGCACATGCCAAGCTGAGTCCTGTTGGGTCATCAGATCTGACGGGG 3536
Our sequence    GCGCCTTGCACATGCCAAGCTGAGTCCTGTTGGGTCATCAGATCTGACGGGG 3536
Score           ****************************************************

JN127373.1      CCCTTTGCCATGGCTTGTCCAAGGGGGACAAGGTAGAACTGGACGTGGCCAT 3588
Our sequence    CCCTTTGCCATGGCTTGTCCAAGGGGGACAAGGTAGAACTGGACGTGGCCAT 3588
Score           ****************************************************

JN127373.1      GGAGGTTTCGGACTTCCGTGGGTCGTCTGGGTCGCCTGTCCTTTGCGACGAG 3640
Our sequence    GGAGGTTTCGGACTTCCGTGGGTCGTCTGGGTCGCCTGTCCTTTGCGACGAG 3640
Score           ****************************************************
```

```
JN127373.1          GGGCACGCAGTAGGAATGCTGGTGTCAGTGCTCCATTCTGGCGGGAGGGTTA  3692
Our sequence        GGGCACGCAGTAGGAATGCTGGTGTCAGTGCTCCATTCTGGCGGGAGGGTTA  3692
Score               ****************************************************

JN127373.1          CGGCTGCAAGGTTCACAAGGCCGTGGACCCAAGTTCCAACAGACGCCAAGAC  3744
Our sequence        CGGCTGCAAGGTTCACAAGGCCGTGGACCCAAGTTCCAACAGACGCCAAGAC  3744
Score               ****************************************************

JN127373.1          CACTACCGAACCCCCGCCGGTGCCGGCAAAAGGAGTTTTCAAAGAGGCCCCG  3796
Our sequence        CACTACCGAACCCCCGCCGGTGCCGGCAAAAGGAGTTTTCAAAGAGGCCCCG  3796
Score               ****************************************************

JN127373.1          TTGTTTATGCCCACGGGGGCCGGGAAGAGCACCCGGGTCCCCCTGGAATATG  3848
Our sequence        TTGTTTATGCCCACGGGGGCCGGGAAGAGCACCCGGGTCCCCCTGGAATATG  3848
Score               ****************************************************

JN127373.1          GCAACATGGGGCACAAGGTCTTGATTCTCAACCCGTCGGTGGCCACCGTGAG  3900
Our sequence        GCAACATGGGGCACAAGGTCTTGATTCTCAACCCGTCGGTGGCCACCGTGAG  3900
Score               ****************************************************

JN127373.1          GGCCATGGGCCCTTACATGGAGAAGCTGGCGGGCAAACATCCAAGCATCTAC  3952
Our sequence        GGCCATGGGCCCTTACATGGAGAAGCTGGCGGGCAAACATCCAAGCATCTAC  3952
Score               ****************************************************

JN127373.1          TGTGGGCACGATACAACTGCCTTCACGAGAATTACTGATTCGCCCCTGACGT  4004
Our sequence        TGTGGGCACGATACAACTGCCTTCACGAGAATTACTGATTCGCCCCTGACGT  4004
Score               ****************************************************
```

```
JN127373.1      ACTCGACCTATGGGAGGTTTCTTGCCAACCCTAGGCAGATGCTACGGGGCGT 4056
Our sequence    ACTCGACCTATGGGAGGTTTCTTGCCAACCCTAGGCAGATGCTACGGGGCGT 4056
Score           ****************************************************

JN127373.1      GTCGGTGGTAATCTGTGATGAGTGCCACAGTCATGACTCAACGGTGTTGCTG 4108
Our sequence    GTCGGTGGTAATCTGTGATGAGTGCCACAGTCATGACTCAACGGTGTTGCTG 4108
Score           ****************************************************

JN127373.1      GGGATTGGGCGCGTCCGGGAGTTGGCTCGTGGGTGTGGGGTACAGCTAGTGC 4160
Our sequence    GGGATTGGGCGCGTCCGGGAGTTGGCTCGTGGGTGTGGGGTACAGCTAGTGC 4160
Score           ****************************************************

JN127373.1      TCTACGCTACAGCCACACCTCCCGGGTCGCCTATGACTCAGCACCCCTCCAT 4212
Our sequence    TCTACGCTACAGCCACACCTCCCGGGTCGCCTATGACTCAGCACCCCTCCAT 4212
Score           ****************************************************

JN127373.1      CATTGAGACAAAGCTGGACGTTGGTGAGATCCCCTTTTATGGGCATGGCATT 4264
Our sequence    CATTGAGACAAAGCTGGACGTTGGTGAGATCCCCTTTTATGGGCATGGCATT 4264
Score           ****************************************************

JN127373.1      CCCTTGGAGCGGATGCGGACCGGCAGACACCTCGTATTTTGTCACTCAAAGG 4316
Our sequence    CCCTTGGAGCGGATGCGGACCGGCAGACACCTCGTATTTTGTCACTCAAAGG 4316
Score           ****************************************************

JN127373.1      CAGAGTGCGAGCGACTTGCTGGCCAGTTCTCTTCCAGGGGGGTCAATGCCAT 4368
Our sequence    CAGAGTGCGAGCGACTTGCTGGCCAGTTCTCTTCCAGGGGGGTCAATGCCAT 4368
Score           ****************************************************
```

```
JN127373.1      TGCTTATTATCGGGGGAAGGACAGTTCCATCATCAAGGACGGTGATCTCGTG 4420
Our sequence    TGCTTATTATCGGGGGAAGGACAGTTCCATCATCAAGGACGGTGATCTCGTG 4420
Score           ****************************************************

JN127373.1      GTTTGCGCTACAGACGCGCTCTCCACGGGGTACACGGGAAACTTCGATTCTG 4472
Our sequence    GTTTGCGCTACAGACGCGCTCTCCACGGGGTACACGGGAAACTTCGATTCTG 4472
Score           ****************************************************

JN127373.1      TCACCGACTGTGGGTTGGTAGTGGAGGAGGTCGTTGAGGTGACCCTTGATCC 4524
Our sequence    TCACCGACTGTGGGTTGGTAGTGGAGGAGGTCGTTGAGGTGACCCTTGATCC 4524
Score           ****************************************************

JN127373.1      CACTATCACCATCTCCCTGCGAACGGTGCCTGCTTCGGCTGAACTGTCGATG 4576
Our sequence    CACTATCACCATCTCCCTGCGAACGGTGCCTGCTTCGGCTGAACTGTCGATG 4576
Score           ****************************************************

JN127373.1      CAAAGGCGAGGACGCACGGGTAGGGGCCGGTCTGGGCGGTACTACTACGCGG 4628
Our sequence    CAAAGGCGAGGACGCACGGGTAGGGGCCGGTCTGGGCGGTACTACTACGCGG 4628
Score           ****************************************************

JN127373.1      GGGTCGGTAAGGCCCCCGCTGGGGTGGTGCGGTCCGGGCCGGTCTGGTCGGC 4680
Our sequence    GGGTCGGTAAGGCCCCCGCTGGGGTGGTGCGGTCCGGGCCGGTCTGGTCGGC 4680
Score           ****************************************************

JN127373.1      GGTGGAAGCTGGAGTGACCTGGTATGGTATGGAGCCTGATCTGACAGCAAAC 4732
Our sequence    GGTGGAAGCTGGAGTGACCTGGTATGGTATGGAGCCTGATCTGACAGCAAAC 4732
Score           ****************************************************
```

```
JN127373.1         CTTCTGAGACTTTACGACGACTGCCCTTACACCGCAGCCGTCGCAGCTGACA 4784
Our sequence       CTTCTGAGACTTTACGACGACTGCCCTTACACCGCAGCCGTCGCAGCTGACA 4784
Score              ****************************************************

JN127373.1         TTGGGGAAGCCGCGGTGTTCTTTGCGGGGCTTGCCCCCCTCAGGATGCATCC 4836
Our sequence       TTGGGGAAGCCGCGGTGTTCTTTGCGGGGCTTGCCCCCCTCAGGATGCATCC 4836
Score              ****************************************************

JN127373.1         CGATGTTAGCTGGGCAAAAGTGCGCGGCGTCAATTGGCCCCTCCTGGTGGGT 4888
Our sequence       CGATGTTAGCTGGGCAAAAGTGCGCGGCGTCAATTGGCCCCTCCTGGTGGGT 4888
Score              ****************************************************

JN127373.1         GTTCAGCGGACCATGTGCCGGGAAACACTGTCTCCCGGACCATCGGATGACC 4940
Our sequence       GTTCAGCGGACCATGTGCCGGGAAACACTGTCTCCCGGACCATCGGATGACC 4940
Score              ****************************************************

JN127373.1         CCCAGTGGGCTGGTCTGAAGGGCCCAAATCCTGTCCCTCTACTGCTGAGGTG 4992
Our sequence       CCCAGTGGGCTGGTCTGAAGGGCCCAAATCCTGTCCCTCTACTGCTGAGGTG 4992
Score              ****************************************************

JN127373.1         GGGCAACGATTTGCCATCCAAAGTGGCCGGCCATCACATTGTGGACGACTTG 5044
Our sequence       GGGCAACGATTTGCCATCCAAAGTGGCCGGCCATCACATTGTGGACGACTTG 5044
Score              ****************************************************

JN127373.1         GTCCGTCGCCTGGGCGTTGCGGAGGGTTATGTCCGCTGCGACGCGGGGCCCA 5096
Our sequence       GTCCGTCGCCTGGGCGTTGCGGAGGGTTATGTCCGCTGCGACGCGGGGCCCA 5096
Score              ****************************************************
```

JN127373.1 TCTTAATGGTGGGCCTCGCTATCGCGGGGGGGATGATCTACGCTTCGTACAC 5148
Our sequence TCTTAATGGTGGGCCTCGCTATCGCGGGGGGGATGATCTACGCTTCGTACAC 5148
Score **

JN127373.1 AGGATCACTCGTGGTGGTAACAGACTGGGATGTAAAGGGGGGTGGCAACCCC 5200
Our sequence AGGATCACTCGTGGTGGTAACAGACTGGGATGTAAAGGGGGGTGGCAACCCC 5200
Score **

JN127373.1 CTTTATCGGAGTGGTGACCAAGCCACCCCGCAGCCCGTCGTGCAGGTGCCCC 5252
Our sequence CTTTATCGGAGTGGTGACCAAGCCACCCCGCAGCCCGTCGTGCAGGTGCCCC 5252
Score **

JN127373.1 CGGTAGACCACCGGCCGGGGGGAGAATCTGCTCCGTCGGACGCCAAGACAGT 5304
Our sequence CGGTAGACCACCGGCCGGGGGGAGAATCTGCTCCGTCGGACGCCAAGACAGT 5304
Score **

JN127373.1 GACAGATGCGGTGGCGGCCATTCAAGTGGACTGTGATTGGTCAGTTATGACC 5356
Our sequence GACAGATGCGGTGGCGGCCATTCAAGTGGACTGTGATTGGTCAGTTATGACC 5356
Score **

JN127373.1 CTGTCGATCGGGGAAGTACTGTCCTTGGCTCAGGCTAAGACTGCCGAGGCCT 5408
Our sequence CTGTCGATCGGGGAAGTACTGTCCTTGGCTCAGGCTAAGACTGCCGAGGCCT 5408
Score **

JN127373.1 ACACAGCAACCGCCAAGTGGCTCGCTGGCTGCTACACGGGGACGCGGGCCGT 5460
Our sequence ACACAGCAACCGCCAAGTGGCTCGCTGGCTGCTACACGGGGACGCGGGCCGT 5460
Score **

```
JN127373.1      CCCCACAGTTTCAATTGTGGACAAGCTCTTTGCCGGCGGGTGGGCGGCGGTG 5512
Our sequence    CCCCACAGTTTCAATTGTGGACAAGCTCTTTGCCGGCGGGTGGGCGGCGGTG 5512
Score           ****************************************************

JN127373.1      GTTGGCCACTGCCACAGCGTCATAGCTGCGGCGGTGGCTGCCTACGGGGCTT 5564
Our sequence    GTTGGCCACTGCCACAGCGTCATAGCTGCGGCGGTGGCTGCCTACGGGGCTT 5564
Score           ****************************************************

JN127373.1      CTAGGAGCCCTCCGTTGGCTGCCGCTGCCTCCTACCTTATGGGATTGGGCGT 5616
Our sequence    CTAGGAGCCCTCCGTTGGCTGCCGCTGCCTCCTACCTTATGGGATTGGGCGT 5616
Score           ****************************************************

JN127373.1      CGGAGGCAATGCTCAGACGCGCTTGGCTTCTGCTCTCCTTCTGGGGGCTGCT 5668
Our sequence    CGGAGGCAATGCTCAGACGCGCTTGGCTTCTGCTCTCCTTCTGGGGGCTGCT 5668
Score           ****************************************************

JN127373.1      GGTACCGCCCTGGGCACTCCCGTCGTTGGTTTGACCATGGCGGGGGCGTTCA 5720
Our sequence    GGTACCGCCCTGGGCACTCCCGTCGTTGGTTTGACCATGGCGGGGGCGTTCA 5720
Score           ****************************************************

JN127373.1      TGGGTGGCGCTAGCGTCTCCCCATCCTTGGTGACCATTTTACTGGGAGCCGT 5772
Our sequence    TGGGTGGCGCTAGCGTCTCCCCATCCTTGGTGACCATTTTACTGGGAGCCGT 5772
Score           ****************************************************

JN127373.1      GGGTGGTTGGGAGGGCGTGGTCAACGCGGCCAGCCTTGTCTTTGACTTCATG 5824
Our sequence    GGGTGGTTGGGAGGGCGTGGTCAACGCGGCCAGCCTTGTCTTTGACTTCATG 5824
Score           ****************************************************
```

```
JN127373.1      GCAGGGAAACTTTCATCAGAGGACCTGTGGTATGCTATCCCGGTGCTCACGA 5876
Our sequence    GCAGGGAAACTTTCATCAGAGGACCTGTGGTATGCTATCCCGGTGCTCACGA 5876
Score           ****************************************************

JN127373.1      GCCCCGGGGCGGGCCTTGCGGGGATCGCTCTTGGACTTGTGTTGTACTCAGC 5928
Our sequence    GCCCCGGGGCGGGCCTTGCGGGGATCGCTCTTGGACTTGTGTTGTACTCAGC 5928
Score           ****************************************************

JN127373.1      CAACAACTCAGGCACTACCACATGGTTGAACCGTCTGCTGACCACGTTGCCG 5980
Our sequence    CAACAACTCAGGCACTACCACATGGTTGAACCGTCTGCTGACCACGTTGCCG 5980
Score           ****************************************************

JN127373.1      CGGTCCTCATGCATCCCCGACAGTTACTTTCAGCAAGCTGACTACTGTGACA 6032
Our sequence    CGGTCCTCATGCATCCCCGACAGTTACTTTCAGCAAGCTGACTACTGTGACA 6032
Score           ****************************************************

JN127373.1      AGGTCTCGGCTGTACTCCGGCGCCTGAGCCTCACACGAACTGTGGTGGCCCT 6084
Our sequence    AGGTCTCGGCTGTACTCCGGCGCCTGAGCCTCACACGAACTGTGGTGGCCCT 6084
Score           ****************************************************

JN127373.1      GGTCAACAGGGAACCAAAAGTCGATGAGGTGCAGGTCGGATACGTCTGGGAT 6136
Our sequence    GGTCAACAGGGAACCAAAAGTCGATGAGGTGCAGGTCGGATACGTCTGGGAT 6136
Score           ****************************************************

JN127373.1      TTGTGGGAGTGGATCATGCGCCAGGTGCGTATGGTGATGGCCAGACTCCGGG 6188
Our sequence    TTGTGGGAGTGGATCATGCGCCAGGTGCGTATGGTGATGGCCAGACTCCGGG 6188
Score           ****************************************************
```

```
JN127373.1      CCCTCTGCCCCGTGGTGTCGTTACCGCTATGGCACTGCGGGGAGGGGTGGTC 6240
Our sequence    CCCTCTGCCCCGTGGTGTCGTTACCGCTATGGCACTGCGGGGAGGGGTGGTC 6240
Score           ****************************************************

JN127373.1      TGGAGAATGGCTGTTGGATGGGCATGTTGAGAGTCGCTGCCTTTGTGGGTGC 6292
Our sequence    TGGAGAATGGCTGTTGGATGGGCATGTTGAGAGTCGCTGCCTTTGTGGGTGC 6292
Score           ****************************************************

JN127373.1      GTCATCACAGGCGACGTGTTTAATGGGCAACTCAAAGAACCAGTTTATTCCA 6344
Our sequence    GTCATCACAGGCGACGTGTTTAATGGGCAACTCAAAGAACCAGTTTATTCCA 6344
Score           ****************************************************

JN127373.1      CAAAGCTGTGCCGACACTATTGGATGGGGACCGTGCCGGTCAACATGCTGGG 6396
Our sequence    CAAAGCTGTGCCGACACTATTGGATGGGGACCGTGCCGGTCAACATGCTGGG 6396
Score           ****************************************************

JN127373.1      ATACGGGGAAACCTCGCCCCTCCTGGCCTCTGACACCCCGAAGGTGGTACCT 6448
Our sequence    ATACGGGGAAACCTCGCCCCTCCTGGCCTCTGACACCCCGAAGGTGGTACCT 6448
Score           ****************************************************

JN127373.1      TTCGGGACGTCGGGCTGGGCGGAGGTGGTGGTGACCCCCACCCACGTGGTGA 6500
Our sequence    TTCGGGACGTCGGGCTGGGCGGAGGTGGTGGTGACCCCCACCCACGTGGTGA 6500
Score           ****************************************************

JN127373.1      TCCGGCGCACCTCGTCCTACAAGTTGTTGCGCCAGCAAATTCTTTCGGCCGC 6552
Our sequence    TCCGGCGCACCTCGTCCTACAAGTTGTTGCGCCAGCAAATTCTTTCGGCCGC 6552
Score           ****************************************************
```

```
JN127373.1      TGTAGCTGAGCCCTACTACGTCGACGGCATTCCGGTCTCATGGGACGCTGAC 6604
Our sequence    TGTAGCTGAGCCCTACTACGTCGACGGCATTCCGGTCTCATGGGACGCTGAC 6604
Score           ****************************************************

JN127373.1      GCGAGAGCACCAGCGATGGTCTATGGCCCCGGGCAAAGTGTTACCATTGACG 6656
Our sequence    GCGAGAGCACCAGCGATGGTCTATGGCCCCGGGCAAAGTGTTACCATTGACG 6656
Score           ****************************************************

JN127373.1      GGGAGCGCTACACCCTGCCGCACCAGCTGCGGCTTAGGAATGTGGCGCCCTC 6708
Our sequence    GGGAGCGCTACACCCTGCCGCACCAGCTGCGGCTTAGGAATGTGGCGCCCTC 6708
Score           ****************************************************

JN127373.1      TGAGGTTTCATCCGAGGTGTCCATTGACATTGGGACGGAGACTGAAGACTCA 6760
Our sequence    TGAGGTTTCATCCGAGGTGTCCATTGACATTGGGACGGAGACTGAAGACTCA 6760
Score           ****************************************************

JN127373.1      GAACTGACTGAGGCCGACTTGCCGCCGGCGGCTGCTGCCCTCCAGGCTATCG 6812
Our sequence    GAACTGACTGAGGCCGACTTGCCGCCGGCGGCTGCTGCCCTCCAGGCTATCG 6812
Score           ****************************************************

JN127373.1      AGAATGCTGCGAGAATTCTTGAGCCTCACATTGATGTCATCATGGAGGACTG 6864
Our sequence    AGAATGCTGCGAGAATTCTTGAGCCTCACATTGATGTCATCATGGAGGACTG 6864
Score           ****************************************************

JN127373.1      CAGTACACCCTCTCTCTGTGGTAGTAGCCGAGAGATGCCAGTGTGGGGCGAA 6916
Our sequence    CAGTACACCCTCTCTCTGTGGTAGTAGCCGAGAGATGCCAGTGTGGGGCGAA 6916
Score           ****************************************************
```

```
JN127373.1       GACATCCCCCGCACTCCATCGCCAGCACTTATCTCGGTTACCGAGAGCAGCT 6968
Our sequence     GACATCCCCCGCACTCCATCGCCAGCACTTATCTCGGTTACCGAGAGCAGCT 6968
Score            ****************************************************

JN127373.1       CAGATGAAAAGACCCCGTCGGTGTCTTCCTCGCAGGAGGATACCCCGTCCTC 7020
Our sequence     CAGATGAAAAGACCCCGTCGGTGTCTTCCTCGCAGGAGGATACCCCGTCCTC 7020
Score            ****************************************************

JN127373.1       TGACTCATTCGAAGTCATTCCAGAGACGGAGACGGCCGGAGGGGATGACAAT 7072
Our sequence     TGACTCATTCGAAGTCATTCCAGAGACGGAGACGGCCGGAGGGGATGACAAT 7072
Score            ****************************************************

JN127373.1       GTCTTCAACGTGGCTCTTTCCGTATTAAAAGCCTTGTTCCCACAGAGCGATG 7124
Our sequence     GTCTTCAACGTGGCTCTTTCCGTATTAAAAGCCTTGTTCCCACAGAGCGATG 7124
Score            ****************************************************

JN127373.1       CCACACGCAAGCTAACGGTGAAGATGTCGTGCTGCGTGGAGAAGAGCGTCAC 7176
Our sequence     CCACACGCAAGCTAACGGTGAAGATGTCGTGCTGCGTGGAGAAGAGCGTCAC 7176
Score            ****************************************************

JN127373.1       GCGCTTCTTTTCCTTGGGGTTGACCGTGGCTGACGTGGCTAGCCTGTGCGAA 7228
Our sequence     GCGCTTCTTTTCCTTGGGGTTGACCGTGGCTGACGTGGCTAGCCTGTGCGAA 7228
Score            ****************************************************

JN127373.1       ATGGAGATCCAGAACCATACAGCCTATTGTGACAAGGTGCGCACTCCGCTTG 7280
Our sequence     ATGGAGATCCAGAACCATACAGCCTATTGTGACAAGGTGCGCACTCCGCTTG 7280
Score            ****************************************************
```

JN127373.1 AATTGCAGGTTGGGTGCTTGGTGGGCAATGAACTTACCTTTGAATGTGACAA 7332
Our sequence AATTGCAGGTTGGGTGCTTGGTGGGCAATGAACTTACCTTTGAATGTGACAA 7332
Score **

JN127373.1 GTGTGAGGCTAGGCAAGAGACCTTGGCTTCTTTCTCCTACATCTGGTCTGGC 7384
Our sequence GTGTGAGGCTAGGCAAGAGACCTTGGCTTCTTTCTCCTACATCTGGTCTGGC 7384
Score **

JN127373.1 GTGCCGCTCACGCGGGCCACTCCGGCCAAGCCCCCTGTGGTGAGGCCGGTTG 7436
Our sequence GTGCCGCTCACGCGGGCCACTCCGGCCAAGCCCCCTGTGGTGAGGCCGGTTG 7436
Score **

JN127373.1 GCTCGTTGTTAGTGGCTGACACCACCAAGGTGTATGTCACCAATCCAGACAA 7488
Our sequence GCTCGTTGTTAGTGGCTGACACCACCAAGGTGTATGTCACCAATCCAGACAA 7488
Score **

JN127373.1 CGTGGGGAGGAGAGTGGACAAGGTTACCTTCTGGCGTGCTCCCAGGGTTCAC 7540
Our sequence CGTGGGGAGGAGAGTGGACAAGGTTACCTTCTGGCGTGCTCCCAGGGTTCAC 7540
Score **

JN127373.1 GACAAATTCCTTGTGGACTCTATCGAGCGCGCGAGAAAGGCAGCTCAAGCCT 7592
Our sequence GACAAATTCCTTGTGGACTCTATCGAGCGCGCGAGAAAGGCAGCTCAAGCCT 7592
Score **

JN127373.1 GCCTAAGCATGGGTTACACTTATGAGGAGGCAATAAGGACTGTTAGGCCACA 7644
Our sequence GCCTAAGCATGGGTTACACTTATGAGGAGGCAATAAGGACTGTTAGGCCACA 7644
Score **

```
JN127373.1      TGCTGCCATGGGCTGGGGATCTAAGGTGTCGGTCAAGGACCTCGCCACCCCT 7696
Our sequence    TGCTGCCATGGGCTGGGGATCTAAGGTGTCGGTCAAGGACCTCGCCACCCCT 7696
Score           ****************************************************

JN127373.1      GCGGGGAAGATGGCTGTCCATGACCGACTTCAGGAGATACTTGAAGGGACTC 7748
Our sequence    GCGGGGAAGATGGCTGTCCATGACCGACTTCAGGAGATACTTGAAGGGACTC 7748
Score           ****************************************************

JN127373.1      CGGTCCCATTTACCCTGACTGTGAAAAAGGAGGTGTTCTTCAAAGACCGTAA 7800
Our sequence    CGGTCCCATTTACCCTGACTGTGAAAAAGGAGGTGTTCTTCAAAGACCGTAA 7800
Score           ****************************************************

JN127373.1      GGAGGAGAAGGCCCCCCGCCTCATTGTGTTCCCTCCCCTGGACTTCCGGATA 7852
Our sequence    GGAGGAGAAGGCCCCCCGCCTCATTGTGTTCCCTCCCCTGGACTTCCGGATA 7852
Score           ****************************************************

JN127373.1      GCTGAAAAGTTAATCCTGGGGGACCCGGGTCGAGTGGCCAAGGCGGTGTTGG 7904
Our sequence    GCTGAAAAGTTAATCCTGGGGGACCCGGGTCGAGTGGCCAAGGCGGTGTTGG 7904
Score           ****************************************************

JN127373.1      GGGGGGCTTACGCCTTCCAGTACACCCCAAACCAGCGGGTTAAGGAGATGCT 7956
Our sequence    GGGGGGCTTACGCCTTCCAGTACACCCCAAACCAGCGGGTTAAGGAGATGCT 7956
Score           ****************************************************

JN127373.1      CAAGCTGTGGGAATCAAAGAAAACCCCTTGCGCCATCTGCGTGGACGCGACG 8008
Our sequence    CAAGCTGTGGGAATCAAAGAAAACCCCTTGCGCCATCTGCGTGGACGCGACG 8008
Score           ****************************************************
```

```
JN127373.1      TGCTTCGACAGTAGCATTACTGAAGAGGACGTGGCGCTGGAGACGGAGCTAT 8060
Our sequence    TGCTTCGACAGTAGCATTACTGAAGAGGACGTGGCGCTGGAGACGGAGCTAT 8060
Score           ****************************************************

JN127373.1      ATGCTCTGGCTTCAGACCACCCAGAGTGGGTGCGAGCCCTGGGGAAATACTA 8112
Our sequence    ATGCTCTGGCTTCAGACCACCCAGAGTGGGTGCGAGCCCTGGGGAAATACTA 8112
Score           ****************************************************

JN127373.1      TGCTTCAGGCACCATGGTTACCCCGGAAGGGGTTCCCGTCGGTGAGAGGTAC 8164
Our sequence    TGCTTCAGGCACCATGGTTACCCCGGAAGGGGTTCCCGTCGGTGAGAGGTAC 8164
Score           ****************************************************

JN127373.1      TGTAGATCCTCAGGGGTTCTGACCACCAGCGCGAGCAACTGCTTGACCTGCT 8216
Our sequence    TGTAGATCCTCAGGGGTTCTGACCACCAGCGCGAGCAACTGCTTGACCTGCT 8216
Score           ****************************************************

JN127373.1      ACATTAAGGTGTCAGCCGCCTGCCAGAGAGTGGGACTGAAAAATGTCTCGCT 8268
Our sequence    ACATTAAGGTGTCAGCCGCCTGCCAGAGAGTGGGACTGAAAAATGTCTCGCT 8268
Score           ****************************************************

JN127373.1      TCTCATCGCGGGCGATGACTGTCTGATCATATGCGAACGGCCAGTGTGCGAC 8320
Our sequence    TCTCATCGCGGGCGATGACTGTCTGATCATATGCGAACGGCCAGTGTGCGAC 8320
Score           ****************************************************

JN127373.1      CCGAGCGAAGCTCTGGGCAGAGCCCTAGCGAGCTATGGGTACGCGTGCGAAC 8372
Our sequence    CCGAGCGAAGCTCTGGGCAGAGCCCTAGCGAGCTATGGGTACGCGTGCGAAC 8372
Score           ****************************************************
```

```
JN127373.1      CTTCGTATCATGCATCATTGGACACGGCCCCCTTCTGCTCCACTTGGCTTGC 8424
Our sequence    CTTCGTATCATGCATCATTGGACACGGCCCCCTTCTGCTCCACTTGGCTTGC 8424
Score           ****************************************************

JN127373.1      TGAGTGCAATGCAGATGGGAAGCGCCATTTCTTCCTGACAACGGACTTTCGG 8476
Our sequence    TGAGTGCAATGCAGATGGGAAGCGCCATTTCTTCCTGACAACGGACTTTCGG 8476
Score           ****************************************************

JN127373.1      AGGCCGCTCGCTCGCATGTCGAGCGAGTACAGTGACCCTATGGCCTCGGCCA 8528
Our sequence    AGGCCGCTCGCTCGCATGTCGAGCGAGTACAGTGACCCTATGGCCTCGGCCA 8528
Score           ****************************************************

JN127373.1      TTGGTTACATCCTCCTTTATCCTTGGCACCCCATCACACGGTGGGTCATCAT 8580
Our sequence    TTGGTTACATCCTCCTTTATCCTTGGCACCCCATCACACGGTGGGTCATCAT 8580
Score           ****************************************************

JN127373.1      CCCGCACGTGCTAACGTGCGCGTTTCGGGGTGGTGGCACACCGTCTGATCCT 8632
Our sequence    CCCGCACGTGCTAACGTGCGCGTTTCGGGGTGGTGGCACACCGTCTGATCCT 8632
Score           ****************************************************

JN127373.1      GTGTGGTGCCAGGTACATGGTAACTACTACAAGTTTCCACTGGACAAACTGC 8684
Our sequence    GTGTGGTGCCAGGTACATGGTAACTACTACAAGTTTCCACTGGACAAACTGC 8684
Score           ****************************************************

JN127373.1      CAAACATCATCGTGGCCCTCCACGGACCAGCAGCGTTGAGGGTTACCGCAGA 8736
Our sequence    CAAACATCATCGTGGCCCTCCACGGACCAGCAGCGTTGAGGGTTACCGCAGA 8736
Score           ****************************************************
```

JN127373.1 CACAACCAAAACAAAAATGGAGGCCGGCAAGGTGCTGAGCGACCTCAAGCTC 8788
Our sequence CACAACCAAAACAAAAATGGAGGCCGGCAAGGTGCTGAGCGACCTCAAGCTC 8788
Score **

JN127373.1 CCGGGCTTAGCGGTCCACCGTAAGAAGGCTGGAGCATTGCGAACCCGCATGC 8840
Our sequence CCGGGCTTAGCGGTCCACCGTAAGAAGGCTGGAGCATTGCGAACCCGCATGC 8840
Score **

JN127373.1 TTCGGTCGCGCGGTTGGGCCGAGTTGGCTCGGGGCCTGTTGTGGCATCCAGG 8892
Our sequence TTCGGTCGCGCGGTTGGGCCGAGTTGGCTCGGGGCCTGTTGTGGCATCCAGG 8892
Score **

JN127373.1 CCTACGGCTCCCCCCCCCCGAGATTGCTGGTATCCCGGGGGGTTTTCCCCTG 8944
Our sequence CCTACGGCTCCCCCCCCCCGAGATTGCTGGTATCCCGGGGGGTTTTCCCCTG 8944
Score **

JN127373.1 TCCCCCCCCTACATGGGGGTGGTTCATCAATTGGATTTCACAAGCCAGAGGA 8996
Our sequence TCCCCCCCCTACATGGGGGTGGTTCATCAATTGGATTTCACAAGCCAGAGGA 8996
Score **

JN127373.1 GTCGCTGGCGGTGGTTGGGGTTCTTAGCCCTGCTCATCGTAGCCCTCTTCGG 9048
Our sequence GTCGCTGGCGGTGGTTGGGGTTCTTAGCCCTGCTCATCGTAGCCCTCTTCGG 9048
Score **

JN127373.1 GTGAACTAAATTCATCTGTTGCGGCAAGGTCTGGTGACTGATCATCACCGGA 9100
Our sequence GTGAACTAAATTCATCTGTTGCGGCAAGGTCTGGTGACTGATCATCACCGGA 9100
Score **

```
JN127373.1      GGAGGTTCCCGCCCTCCCCGCCCCAGGGGTCTCCCCGCTGGGTAAAAAGGGC 9152
Our sequence    GGAGGTTCCCGCCCTCCCCGCCCCAGGGGTCTCCCCGCTGGGTAAAAAGGGC 9152
Score           ****************************************************

JN127373.1      CCGGCCTTGGGAGGCATGGTGGTTACTAACCCCCTGGCAGGGTCAAAGCCTG 9204
Our sequence    CCGGCCTTGGGAGGCATGGTGGTTACTAACCCCCTGGCAGGGTCAAAGCCTG 9204
Score           ****************************************************

JN127373.1      ATGGTGCTAATGCACTGCCACTTCGGTGGCGGGTCGCTACCTTATAGCGTAA 9256
Our sequence    ATGGTGCTAATGCACTGCCACTTCGGTGGCGGGTCGCTACCTTATAGCGTAA 9256
Score           ****************************************************

JN127373.1      TCCGTGACTACGGGCTGCTCGCAGAGCCCTCCCCGGATGGGGCACAGTGCAC 9308
Our sequence    TCCGTGACTACGGGCTGCTCGCAGAGCCCTCCCCGGATGGGGCACAGTGCAC 9308
Score           ****************************************************

JN127373.1      TGTGATCTGAAGGGGTGCACCCCGGTAAGAGCTCAG 9344
Our sequence    TGTGATCTGAAGGGGTGCACCCCGGTAAGAGCTCAG 9344
Score           ************************************
```
