# Supplementary material for: Whole-genome sequencing of human Pegivirus variant from an Egyptian patient co-infected with hepatitis C virus: a case report
Source: Virol J. 2019 Nov 11;16:132. doi: 10.1186/s12985-019-1242-5 (PMC6849219; doi:10.1186/s12985-019-1242-5)
Supplement: Supplementary file 3 — Additional file 3: Identification of 808 SNPs. [file 12985_2019_1242_MOESM3_ESM.docx]

**Supplementary file 4:**

Ref Genom Type

136 110 SNP A G Transition

306 280 SNP T C Transition

320 294 SNP C T Transition

396 370 SNP G A Transition

406 380 SNP A G Transition

409 383 SNP G A Transition

416 390 SNP G A Transition

425 399 SNP C T Transition

431 405 SNP C T Transition

436 410 SNP T A Transversion

438 412 SNP C T Transition

542 516 SNP G T Transversion

543 517 SNP T A Transversion

546 520 SNP T C Transition

547 521 SNP A G Transition

572 546 SNP C T Transition

619 593 SNP C T Transition

620 594 SNP A C Transversion

622 596 SNP A G Transition

625 599 SNP C T Transition

626 600 SNP A G Transition

628 602 SNP G C Transversion

630 604 SNP A G Transition

646 620 SNP A G Transition

649 623 SNP T C Transition

655 629 SNP C T Transition

660 634 SNP C T Transition

682 656 SNP T G Transversion

685 659 SNP G A Transition

688 662 SNP C T Transition

706 680 SNP A G Transition

709 683 SNP T G Transversion

712 686 SNP C T Transition

724 698 SNP C T Transition

781 755 SNP G A Transition

796 770 SNP T G Transversion

799 773 SNP A G Transition

802 776 SNP G T Transversion

811 785 SNP C T Transition

813 787 SNP A T Transversion

823 797 SNP G A Transition

829 803 SNP C G Transversion

832 806 SNP T C Transition

835 809 SNP T C Transition

841 815 SNP A G Transition

847 821 SNP G T Transversion

856 830 SNP A G Transition

859 833 SNP T C Transition

874 848 SNP T G Transversion

877 851 SNP G A Transition

880 854 SNP G C Transversion

887 861 SNP A G Transition

892 866 SNP T G Transversion

895 869 SNP T C Transition

912 886 SNP C T Transition

920 894 SNP A G Transition

928 902 SNP T C Transition

964 938 SNP A G Transition

988 962 SNP A G Transition

992 966 SNP C T Transition

1003 977 SNP C T Transition

1006 980 SNP T C Transition

1024 998 SNP T C Transition

1033 1007 SNP G A Transition

1036 1010 SNP G A Transition

1045 1019 SNP C T Transition

1051 1025 SNP T G Transversion

1058 1032 SNP G A Transition

1073 1047 SNP T C Transition

1085 1059 SNP T G Transversion

1096 1070 SNP C A Transversion

1105 1079 SNP T A Transversion

1111 1085 SNP G A Transition

1114 1088 SNP A G Transition

1120 1094 SNP C T Transition

1126 1100 SNP T G Transversion

1168 1142 SNP G C Transversion

1171 1145 SNP C T Transition

1177 1151 SNP G C Transversion

1180 1154 SNP C T Transition

1210 1184 SNP A C Transversion

1222 1196 SNP T C Transition

1234 1208 SNP G A Transition

1247 1221 SNP A G Transition

1249 1223 SNP A G Transition

1261 1235 SNP A G Transition

1273 1247 SNP C A Transversion

1276 1250 SNP A T Transversion

1309 1283 SNP T C Transition

1312 1286 SNP T C Transition

1321 1295 SNP G A Transition

1325 1299 SNP A T Transversion

1327 1301 SNP C G Transversion

1334 1308 SNP C G Transversion

1345 1319 SNP G A Transition

1346 1320 SNP A G Transition

1348 1322 SNP T C Transition

1351 1325 SNP T C Transition

1384 1358 SNP G A Transition

1405 1379 SNP A G Transition

1414 1388 SNP G A Transition

1420 1394 SNP C T Transition

1423 1397 SNP C T Transition

1429 1403 SNP T C Transition

1441 1415 SNP T C Transition

1453 1427 SNP C T Transition

1471 1445 SNP T C Transition

1480 1454 SNP T C Transition

1486 1460 SNP T C Transition

1494 1468 SNP A G Transition

1501 1475 SNP C T Transition

1509 1483 SNP G A Transition

1510 1484 SNP T C Transition

1519 1493 SNP A G Transition

1525 1499 SNP C A Transversion

1528 1502 SNP T A Transversion

1534 1508 SNP T C Transition

1537 1511 SNP T C Transition

1549 1523 SNP A T Transversion

1552 1526 SNP G C Transition

1555 1529 SNP G T Transversion

1564 1538 SNP C T Transition

1570 1544 SNP C T Transition

1571 1545 SNP A G Transition

1572 1546 SNP C T Transition

1576 1550 SNP G A Transition

1588 1562 SNP C T Transition

1597 1571 SNP A G Transition

1598 1572 SNP G A Transition

1600 1574 SNP G A Transition

1606 1580 SNP T C Transition

1608 1582 SNP C T Transition

1609 1583 SNP T G Transversion

1660 1634 SNP T C Transition

1675 1649 SNP T C Transition

1681 1655 SNP G A Transition

1684 1658 SNP A T Transversion

1685 1659 SNP C A Transversion

1693 1667 SNP A T Transversion

1705 1679 SNP C T Transition

1714 1688 SNP A C Transversion

1726 1700 SNP A T Transversion

1744 1718 SNP G A Transition

1756 1730 SNP T C Transition

1762 1736 SNP G A Transition

1772 1746 SNP G A Transition

1777 1751 SNP C T Transition

1780 1754 SNP G A Transition

1792 1766 SNP C A Transversion

1793 1767 SNP G A Transition

1794 1768 SNP T A Transversion

1810 1784 SNP C T Transition

1846 1820 SNP T C Transition

1864 1838 SNP T C Transition

1867 1841 SNP C T Transition

1870 1844 SNP G A Transition

1889 1863 SNP G A Transition

1894 1868 SNP G A Transition

1897 1871 SNP C T Transition

1900 1874 SNP T A Transversion

1905 1879 SNP C A Transversion

1912 1886 SNP C T Transition

1921 1895 SNP T C Transition

1933 1907 SNP C T Transition

1939 1913 SNP A G Transition

1942 1916 SNP T G Transversion

1948 1922 SNP T C Transition

1957 1931 SNP T C Transition

1972 1946 SNP C T Transition

1993 1967 SNP A T Transversion

2003 1977 SNP C T Transition

2005 1979 SNP T G Transversion

2059 2033 SNP T C Transition

2062 2036 SNP T C Transition

2071 2045 SNP C T Transition

2074 2048 SNP C T Transition

2110 2084 SNP C T Transition

2116 2090 SNP C T Transition

2125 2099 SNP C T Transition

2131 2105 SNP A G Transition

2152 2126 SNP A G Transition

2164 2138 SNP C T Transition

2170 2144 SNP G C Transversion

2180 2154 SNP T C Transition

2185 2159 SNP T C Transition

2191 2165 SNP C T Transition

2209 2183 SNP G A Transition

2222 2196 SNP T C Transition

2242 2216 SNP C T Transition

2246 2220 SNP T C Transition

2248 2222 SNP G C Transversion

2251 2225 SNP C A Transversion

2263 2237 SNP G A Transition

2286 2260 SNP T C Transition

2287 2261 SNP A G Transition

2290 2264 SNP T C Transition

2293 2267 SNP T C Transition

2304 2278 SNP T C Transition

2323 2297 SNP T G Transversion

2326 2300 SNP T G Transversion

2348 2322 SNP T C Transition

2359 2333 SNP C T Transition

2360 2334 SNP T C Transition

2365 2339 SNP A C Transversion

2377 2351 SNP A G Transition

2380 2354 SNP T C Transition

2386 2360 SNP A C Transversion

2392 2366 SNP A T Transversion

2393 2367 SNP T C Transition

2395 2369 SNP G A Transition

2398 2372 SNP T A Transversion

2404 2378 SNP A G Transition

2413 2387 SNP T C Transition

2428 2402 SNP C G Transversion

2431 2405 SNP T C Transition

2446 2420 SNP C T Transition

2455 2429 SNP A G Transition

2473 2447 SNP G A Transversion

2476 2450 SNP T C Transition

2536 2510 SNP A C Transversion

2554 2528 SNP C T Transition

2574 2548 SNP T C Transition

2575 2549 SNP G T Transversion

2581 2555 SNP G T Transversion

2608 2582 SNP G A Transition

2623 2597 SNP A G Transition

2626 2600 SNP C T Transition

2630 2604 SNP G A Transition

2635 2609 SNP G A Transition

2641 2615 SNP C T Transition

2647 2621 SNP G A Transition

2688 2662 SNP G A Transition

2692 2666 SNP A G Transition

2695 2669 SNP C T Transition

2702 2676 SNP A G Transition

2707 2681 SNP C T Transition

2722 2696 SNP G C Transition

2743 2717 SNP A G Transition

2749 2723 SNP C T Transition

2755 2729 SNP C T Transition

2761 2735 SNP C T Transition

2767 2741 SNP C T Transition

2773 2747 SNP T C Transition

2774 2748 SNP C T Transition

2782 2756 SNP A G Transition

2800 2774 SNP A G Transition

2818 2792 SNP C G Transition

2821 2795 SNP C T Transition

2833 2807 SNP G C Transition

2858 2832 SNP C T Transition

2866 2840 SNP G A Transition

2876 2850 SNP T C Transition

2884 2858 SNP C T Transition

2903 2877 SNP C T Transition

2908 2882 SNP G T Transition

2923 2897 SNP C T Transition

2931 2905 SNP T C Transition

2935 2909 SNP G T Transversion

2941 2915 SNP G A Transition

2956 2930 SNP A G Transition

2977 2951 SNP T C Transition

2983 2957 SNP A G Transition

2988 2962 SNP C T Transition

2998 2972 SNP T C Transition

3004 2978 SNP C T Transition

3013 2987 SNP T C Transition

3023 2997 SNP G T Transversion

3025 2999 SNP A T Transversion

3038 3012 SNP C T Transition

3040 3014 SNP A G Transition

3052 3026 SNP T C Transition

3058 3032 SNP C T Transition

3070 3044 SNP A G Transition

3076 3050 SNP T C Transition

3103 3077 SNP G A Transition

3106 3080 SNP C T Transition

3124 3098 SNP C G Transition

3151 3125 SNP T C Transition

3160 3134 SNP T A Transversion

3163 3137 SNP C T Transition

3166 3140 SNP T C Transition

3169 3143 SNP T C Transition

3175 3149 SNP A G Transition

3178 3152 SNP T G Transversion

3179 3153 SNP T C Transition

3184 3158 SNP C A Transversion

3187 3161 SNP C T Transition

3190 3164 SNP T C Transition

3196 3170 SNP G A Transition

3229 3203 SNP G A Transition

3241 3215 SNP A T Transversion

3250 3224 SNP A G Transition

3259 3233 SNP A G Transition

3286 3260 SNP T A Transversion

3310 3284 SNP C T Transition

3316 3290 SNP T C Transition

3343 3317 SNP A G Transition

3346 3320 SNP C T Transition

3352 3326 SNP A G Transition

3364 3338 SNP G C Transversion

3367 3341 SNP G A Transition

3370 3344 SNP C T Transition

3371 3345 SNP T C Transition

3373 3347 SNP A G Transition

3376 3350 SNP T C Transition

3380 3354 SNP C T Transition

3391 3365 SNP G T Transversion

3394 3368 SNP A T Transversion

3460 3434 SNP A G Transition

3475 3449 SNP A G Transition

3487 3461 SNP C T Transition

3490 3464 SNP T C Transition

3496 3470 SNP T C Transition

3511 3485 SNP G A Transition

3512 3486 SNP A G Transition

3515 3489 SNP T C Transition

3523 3497 SNP T A Transversion

3529 3503 SNP G A Transition

3532 3506 SNP G T Transversion

3550 3524 SNP T C Transition

3553 3527 SNP G A Transition

3556 3530 SNP C T Transition

3565 3539 SNP T C Transition

3592 3566 SNP T C Transition

3598 3572 SNP C A Transversion

3619 3593 SNP A G Transition

3622 3596 SNP G T Transversion

3625 3599 SNP T G Transversion

3643 3617 SNP C T Transition

3658 3632 SNP G T Transversion

3682 3656 SNP C A Transversion

3688 3662 SNP T G Transversion

3700 3674 SNP A C Transversion

3709 3683 SNP G C Transversion

3712 3686 SNP A G Transition

3718 3692 SNP C T Transition

3724 3698 SNP A T Transversion

3727 3701 SNP C A Transversion

3730 3704 SNP A G Transition

3737 3711 SNP C A Transversion

3754 3728 SNP A T Transversion

3757 3731 SNP G A Transition

3758 3732 SNP G A Transition

3769 3743 SNP A G Transition

3775 3749 SNP C T Transition

3778 3752 SNP T C Transition

3814 3788 SNP G A Transition

3824 3798 SNP C T Transition

3829 3803 SNP C T Transition

3835 3809 SNP T C Transition

3844 3818 SNP T C Transition

3859 3833 SNP A G Transition

3866 3840 SNP T C Transition

3871 3845 SNP G A Transition

3874 3848 SNP C T Transition

3880 3854 SNP T C Transition

3886 3860 SNP C G Transversion

3901 3875 SNP C T Transition

3904 3878 SNP G C Transversion

3910 3884 SNP C G Transversion

3919 3893 SNP T C Transition

3922 3896 SNP T C Transition

3950 3924 SNP C A Transversion

3951 3925 SNP G A Transition

3961 3935 SNP T C Transition

3970 3944 SNP C A Transversion

3973 3947 SNP T C Transition

3985 3959 SNP A G Transition

3994 3968 SNP G A Transition

3997 3971 SNP A T Transversion

4009 3983 SNP G A Transition

4012 3986 SNP C T Transition

4015 3989 SNP C T Transition

4018 3992 SNP C T Transition

4021 3995 SNP A G Transition

4025 3999 SNP T C Transition

4030 4004 SNP C G Transversion

4036 4010 SNP T G Transversion

4042 4016 SNP C T Transition

4074 4047 SNP T C Transition

4076 4049 SNP G A Transition

4079 4052 SNP A G Transition

4082 4055 SNP T C Transition

4085 4058 SNP C G Transversion

4124 4097 SNP T A Transversion

4127 4100 SNP A G Transition

4152 4125 SNP A C Transversion

4157 4130 SNP A G Transition

4158 4131 SNP C T Transition

4166 4139 SNP G T Transversion

4175 4148 SNP A G Transition

4178 4151 SNP G A Transition

4181 4154 SNP A G Transition

4184 4157 SNP T A Transversion

4199 4172 SNP G A Transition

4205 4178 SNP G A Transition

4211 4184 SNP T C Transition

4214 4187 SNP A G Transition

4226 4199 SNP C T Transition

4235 4208 SNP T C Transition

4238 4211 SNP A C Transversion

4259 4232 SNP T C Transition

4262 4235 SNP G T Transversion

4265 4238 SNP A T Transversion

4286 4259 SNP C T Transition

4296 4269 SNP C T Transition

4319 4292 SNP G A Transition

4325 4298 SNP T C Transition

4331 4304 SNP C T Transition

4334 4307 SNP C T Transition

4337 4310 SNP T C Transition

4340 4313 SNP T A Transversion

4346 4319 SNP G A Transition

4358 4331 SNP G A Transition

4364 4337 SNP C T Transition

4367 4340 SNP T C Transition

4373 4346 SNP T C Transition

4376 4349 SNP C T Transition

4388 4361 SNP T C Transition

4397 4370 SNP A T Transversion

4400 4373 SNP C T Transition

4403 4376 SNP C T Transition

4407 4380 SNP A C Transversion

4412 4385 SNP C G Transversion

4415 4388 SNP A G Transition

4433 4406 SNP A G Transition

4436 4409 SNP T C Transition

4439 4412 SNP A T Transversion

4442 4415 SNP C T Transition

4445 4418 SNP A C Transversion

4460 4433 SNP T A Transversion

4472 4445 SNP T C Transition

4475 4448 SNP T G Transversion

4487 4460 SNP G A Transition

4490 4463 SNP T C Transition

4496 4469 SNP C T Transition

4499 4472 SNP C T Transition

4514 4487 SNP T G Transversion

4517 4490 SNP A G Transition

4520 4493 SNP G A Transition

4556 4529 SNP C T Transition

4559 4532 SNP A C Transversion

4574 4547 SNP T A Transversion

4583 4556 SNP C T Transition

4586 4559 SNP G T Transversion

4592 4565 SNP A T Transversion

4595 4568 SNP G A Transition

4596 4569 SNP T C Transition

4598 4571 SNP A G Transition

4607 4580 SNP G A Transition

4613 4586 SNP G A Transition

4661 4634 SNP G C Transition

4664 4637 SNP C T Transition

4667 4640 SNP A G Transition

4673 4646 SNP T C Transition

4679 4652 SNP T G Transversion

4697 4670 SNP C G Transversion

4715 4688 SNP G A Transition

4718 4691 SNP C T Transition

4742 4715 SNP A G Transition

4748 4721 SNP C T Transition

4749 4722 SNP T C Transition

4754 4727 SNP T A Transversion

4802 4775 SNP T C Transition

4841 4814 SNP T G Transversion

4844 4817 SNP C T Transition

4847 4820 SNP G C Transversion

4853 4826 SNP T C Transition

4856 4829 SNP A G Transition

4865 4838 SNP T C Transition

4871 4844 SNP C T Transition

4874 4847 SNP T C Transition

4886 4859 SNP T G Transversion

4895 4868 SNP T C Transition

4898 4871 SNP C T Transition

4964 4937 SNP C T Transition

4979 4952 SNP A T Transversion

4994 4967 SNP G A Transition

5000 4973 SNP C T Transition

5006 4979 SNP A T Transversion

5027 5000 SNP T C Transition

5063 5036 SNP A G Transition

5070 5043 SNP C T Transition

5090 5063 SNP G T Transversion

5093 5066 SNP T G Transversion

5114 5087 SNP T C Transition

5120 5093 SNP T G Transversion

5129 5102 SNP G A Transition

5138 5111 SNP G C Transversion

5141 5114 SNP T C Transition

5147 5120 SNP T C Transition

5153 5126 SNP C G Transversion

5156 5129 SNP C G Transversion

5168 5141 SNP G T Transversion

5171 5144 SNP A G Transition

5174 5147 SNP T C Transition

5195 5168 SNP T A Transversion

5210 5183 SNP G A Transition

5225 5198 SNP T C Transition

5243 5216 SNP C T Transition

5249 5222 SNP G A Transversion

5258 5231 SNP A G Transition

5261 5234 SNP A G Transition

5267 5240 SNP T C Transition

5276 5249 SNP C G Transversion

5279 5252 SNP T C Transition

5291 5264 SNP T C Transition

5303 5276 SNP G A Transition

5312 5285 SNP A T Transversion

5315 5288 SNP A G Transition

5316 5289 SNP G T Transversion

5321 5294 SNP T C Transition

5324 5297 SNP T C Transition

5348 5321 SNP A G Transition

5357 5330 SNP G A Transition

5366 5339 SNP C T Transition

5426 5399 SNP C T Transition

5439 5412 SNP T A Transversion

5495 5468 SNP T A Transversion

5525 5498 SNP T C Transition

5528 5501 SNP A G Transition

5537 5510 SNP C G Transversion

5543 5516 SNP A T Transversion

5552 5525 SNP T C Transition

5558 5531 SNP T C Transition

5561 5534 SNP A C Transversion

5573 5546 SNP A G Transition

5582 5555 SNP T C Transition

5585 5558 SNP T C Transition

5594 5567 SNP C T Transition

5597 5570 SNP A G Transition

5600 5573 SNP T C Transition

5603 5576 SNP C T Transition

5612 5585 SNP G T Transversion

5615 5588 SNP T C Transition

5618 5591 SNP G T Transversion

5627 5600 SNP T C Transition

5630 5603 SNP C T Transition

5636 5609 SNP C A Transversion

5654 5627 SNP C T Transition

5666 5639 SNP T C Transition

5667 5640 SNP C T Transition

5675 5648 SNP G T Transversion

5684 5657 SNP C T Transition

5685 5658 SNP T C Transition

5699 5672 SNP A T Transversion

5702 5675 SNP T C Transition

5705 5678 SNP T C Transition

5711 5684 SNP T C Transition

5717 5690 SNP T C Transition

5723 5696 SNP G T Transversion

5726 5699 SNP C T Transition

5729 5702 SNP A G Transition

5744 5717 SNP A G Transition

5753 5726 SNP C T Transition

5756 5729 SNP G C Transversion

5759 5732 SNP C T Transition

5762 5735 SNP T C Transition

5765 5738 SNP T C Transition

5780 5753 SNP C G Transversion

5786 5759 SNP C T Transition

5790 5763 SNP T C Transition

5795 5768 SNP G A Transition

5825 5798 SNP T C Transition

5831 5804 SNP T C Transition

5864 5837 SNP A T Transversion

5888 5861 SNP C T Transition

5903 5876 SNP C G Transversion

5909 5882 SNP G C Transversion

5933 5906 SNP C T Transition

5936 5909 SNP A T Transversion

5939 5912 SNP G A Transition

5942 5915 SNP G T Transversion

5957 5930 SNP A C Transversion

5966 5939 SNP T A Transversion

5975 5948 SNP T C Transition

6014 5987 SNP T C Transition

6017 5990 SNP G A Transition

6044 6017 SNP G A Transition

6047 6020 SNP A T Transversion

6053 6026 SNP T C Transition

6065 6038 SNP A C Transversion

6068 6041 SNP A G Transition

6071 6044 SNP C T Transition

6074 6047 SNP G A Transition

6080 6053 SNP A G Transition

6095 6068 SNP G A Transition

6128 6101 SNP T A Transversion

6131 6104 SNP G A Transition

6134 6107 SNP T C Transition

6137 6110 SNP C T Transition

6143 6116 SNP A G Transition

6149 6122 SNP T C Transition

6164 6137 SNP C T Transition

6173 6146 SNP A G Transition

6188 6161 SNP A G Transition

6194 6167 SNP C T Transition

6198 6171 SNP T G Transversion

6218 6191 SNP T C Transition

6236 6209 SNP A G Transition

6237 6210 SNP C T Transition

6242 6215 SNP C G Transversion

6245 6218 SNP G A Transition

6269 6242 SNP C T Transition

6290 6263 SNP C G Transversion

6294 6267 SNP A G Transition

6311 6284 SNP A T Transversion

6339 6312 SNP C T Transition

6341 6314 SNP A T Transversion

6344 6317 SNP C T Transition

6425 6398 SNP C A Transversion

6428 6401 SNP T C Transition

6434 6407 SNP G A Transition

6443 6416 SNP T C Transition

6447 6420 SNP T C Transition

6455 6428 SNP C T Transition

6479 6452 SNP T C Transition

6488 6461 SNP T G Transversion

6497 6470 SNP T G Transversion

6509 6482 SNP T G Transversion

6515 6488 SNP T C Transition

6518 6491 SNP T C Transition

6530 6503 SNP T C Transition

6531 6504 SNP A C Transversion

6533 6506 SNP A G Transition

6536 6509 SNP G C Transversion

6539 6512 SNP G C Transversion

6570 6543 SNP T C Transition

6572 6545 SNP A T Transversion

6590 6563 SNP A G Transition

6599 6572 SNP T C Transition

6602 6575 SNP T C Transition

6620 6593 SNP G A Transition

6625 6598 SNP G A Transition

6626 6599 SNP T C Transition

6629 6602 SNP C T Transition

6636 6609 SNP C A Transversion

6641 6614 SNP G A Transition

6644 6617 SNP G A Transition

6647 6620 SNP C G Transversion

6662 6635 SNP T C Transition

6710 6683 SNP A G Transition

6806 6779 SNP T C Transition

6815 6788 SNP A G Transition

6818 6791 SNP A G Transition

6829 6802 SNP C T Transition

6839 6812 SNP T C Transition

6854 6827 SNP G A Transition

6860 6833 SNP C T Transition

6866 6839 SNP C T Transition

6869 6842 SNP T C Transition

6911 6884 SNP C T Transition

6932 6905 SNP T A Transversion

6940 6913 SNP A G Transition

6941 6914 SNP T C Transition

6950 6923 SNP T C Transition

6972 6945 SNP T C Transition

6974 6947 SNP G T Transversion

6977 6950 SNP A C Transversion

6993 6966 SNP T A Transversion

6995 6968 SNP T C Transition

7022 6995 SNP C T Transition

7025 6998 SNP T C Transition

7050 7023 SNP C G Transversion

7052 7025 SNP T C Transition

7054 7027 SNP T C Transition

7067 7040 SNP C T Transition

7071 7044 SNP A G Transition

7080 7053 SNP C A Transversion

7083 7056 SNP T G Transversion

7084 7057 SNP T C Transition

7087 7060 SNP A G Transition

7088 7061 SNP G A Transition

7094 7067 SNP G T Transversion

7097 7070 SNP A C Transversion

7099 7072 SNP G A Transition

7100 7073 SNP C T Transition

7121 7094 SNP T C Transition

7139 7112 SNP T C Transition

7140 7113 SNP T C Transition

7160 7133 SNP T C Transition

7181 7154 SNP T G Transversion

7215 7188 SNP C T Transition

7238 7211 SNP T C Transition

7246 7219 SNP A G Transition

7253 7226 SNP T C Transition

7254 7227 SNP A G Transition

7319 7292 SNP C T Transition

7346 7319 SNP T C Transition

7382 7355 SNP T C Transition

7388 7361 SNP G T Transversion

7391 7364 SNP C T Transition

7403 7376 SNP T C Transition

7419 7392 SNP A C Transversion

7421 7394 SNP T C Transition

7424 7397 SNP T G Transversion

7425 7398 SNP A C Transversion

7433 7406 SNP C T Transition

7439 7412 SNP T C Transition

7442 7415 SNP A G Transition

7463 7436 SNP G T Transversion

7469 7442 SNP A G Transition

7481 7454 SNP C T Transition

7517 7490 SNP T C Transition

7523 7496 SNP T G Transversion

7532 7505 SNP C G Transversion

7541 7514 SNP A T Transversion

7556 7529 SNP A T Transversion

7559 7532 SNP A C Transversion

7562 7535 SNP A G Transition

7568 7541 SNP T C Transition

7577 7550 SNP T C Transition

7580 7553 SNP C T Transition

7586 7559 SNP T C Transition

7589 7562 SNP C T Transition

7592 7565 SNP T C Transition

7601 7574 SNP C G Transversion

7604 7577 SNP G A Transition

7610 7583 SNP T A Transversion

7664 7637 SNP A T Transversion

7706 7679 SNP T C Transition

7713 7686 SNP T C Transition

7715 7688 SNP G C Transversion

7751 7724 SNP G A Transition

7823 7796 SNP T C Transition

7835 7808 SNP A G Transition

7890 7863 SNP C T Transition

7892 7865 SNP C A Transversion

7943 7916 SNP T C Transition

7975 7948 SNP G A Transition

7991 7964 SNP A G Transition

7997 7970 SNP G A Transition

8009 7982 SNP G C Transversion

8012 7985 SNP A T Transversion

8024 7997 SNP T C Transition

8030 8003 SNP T C Transition

8051 8024 SNP T C Transition

8060 8033 SNP G A Transition

8069 8042 SNP T G Transversion

8072 8045 SNP C G Transversion

8081 8054 SNP A G Transition

8087 8060 SNP T A Transversion

8090 8063 SNP C T Transition

8094 8067 SNP T C Transition

8108 8081 SNP T C Transition

8123 8096 SNP G A Transition

8144 8117 SNP C T Transition

8147 8120 SNP T A Transversion

8153 8126 SNP T C Transition

8159 8132 SNP C T Transition

8162 8135 SNP T C Transition

8174 8147 SNP G T Transversion

8180 8153 SNP G C Transversion

8183 8156 SNP C T Transition

8198 8171 SNP G A Transition

8207 8180 SNP T G Transversion

8211 8184 SNP T C Transition

8225 8198 SNP A G Transition

8231 8204 SNP T C Transition

8234 8207 SNP T C Transition

8249 8222 SNP C T Transition

8297 8270 SNP C T Transition

8303 8276 SNP T C Transition

8306 8279 SNP A G Transition

8309 8282 SNP T C Transition

8334 8307 SNP A C Transversion

8345 8318 SNP T C Transition

8348 8321 SNP T C Transition

8351 8324 SNP T G Transversion

8375 8348 SNP T A Transversion

8378 8351 SNP C G Transversion

8384 8357 SNP C T Transition

8453 8426 SNP C T Transition

8465 8438 SNP G A Transition

8492 8465 SNP T A Transversion

8495 8468 SNP T G Transversion

8510 8483 SNP T G Transversion

8528 8501 SNP T C Transition

8534 8507 SNP T C Transition

8549 8522 SNP T C Transition

8579 8552 SNP C T Transition

8609 8582 SNP T C Transition

8624 8597 SNP A G Transition

8630 8603 SNP A G Transition

8675 8648 SNP T A Transversion

8684 8657 SNP T C Transition

8696 8669 SNP C T Transition

8771 8744 SNP T C Transition

8825 8798 SNP G A Transition

8837 8810 SNP A T Transversion

8852 8825 SNP G A Transition

8861 8834 SNP A C Transversion

8870 8843 SNP C T Transition

8888 8861 SNP T C Transition

8892 8865 SNP C T Transition

8894 8867 SNP A G Transition

8939 8912 SNP T C Transition

8957 8930 SNP A G Transition

8984 8957 SNP T C Transition

9011 8984 SNP T C Transition

9057 9030 SNP T C Transition

9065 9038 SNP G A Transition

9116 9089 SNP C G Transversion

9125 9098 SNP T C Transition

9172 9145 SNP G A Transition

9224 9197 SNP T C Transition

9255 9228 SNP A T Transversion

9371 9344 SNP G A Transition
