# Supplementary material for: Whole-genome sequencing of human Pegivirus variant from an Egyptian patient co-infected with hepatitis C virus: a case report
Source: Virol J. 2019 Nov 11;16:132. doi: 10.1186/s12985-019-1242-5 (PMC6849219; doi:10.1186/s12985-019-1242-5)
Supplement: Supplementary file 4 — Additional file 4: Alignments of the NS5A protein structure of HCV and HPgV. [file 12985_2019_1242_MOESM4_ESM.pdf]

```
HcV-NS5A            ---------------------------PSQTPSQVDQRRLFHPVRHILVTGGLGL 28
HGV-NS5A-2          NQKSMRCRSDTSGICGSGSCARCVW*WPDSGPSAPWCRYRYGTAGRGGLENGCWM 55
Score                                          *.. **    *  : .. :  : .*  :

HcV-NS5A            GLHRTQ*LQDVAQSKTAASHARHPLPFLPEGI*GGMARGWCNAHHMLLRG-RNSR 82
HGV-NS5A-2          GMLRVAAFVGASSQATCLMGNSKNQ-FIPQSCADTIGWGPCRSTCWDTGKPRPSW 109
Score               *: *.  : ..:.. *.     :   *:*:.  . :. * *.:        * *

HcV-NS5A            PHQER---LHEDRGAEHLQQHLARYVPHQRLHHRSWRAHPVAELQVCDVAGVC*G 134
HGV-NS5A-2          PLTPRRWYLSGRRAGRRWW*PPPTW*SGAPRPTSCCASKFFRPL*LSPTTSTAFR 164
Score               *   *   *   *...:     . : .       .  :: .  * :. .:...

HcV-NS5A            VRGSA*GRRLPLHHWGDAG*HQVPLPSSCS*VLHRGGRRQAPSVRPPMQALAEGG 189
HGV-NS5A-2          SHGTLTR---EHQRWSMAPGKVLPLTGSATPCRTSCGLGMWRPLRFHPRCPLTLG 216
Score                :*:        ::*. *  : :**..*.:      *     .:*   :.    *

HcV-NS5A            GDIFSGPQHIRGGIPAPMRAGARRKCANIHANRSISYNSRSSRSPVGQGITT-LP 243
HGV-NS5A-2          RRLKTQN*-LRPTCRRRLLPSRLSRMLREFLSLTLMSSWRTAVHPLSVVVAERCQ 270
Score                 : :    :*      : ..   :  . . . ::  . *::  *:.  ::

HcV-NS5A            GQFLREPIVCSIS*SHMHPSS*FPWR*---PHRV*PLMGY*CYMLLYRG*V*GEG 295
HGV-NS5A-2          CGAKTSPALHRQHLSRLPRAAQMKRPRRCLPRRRIPRPLTHSKSFQRRRRPEGMT 325
Score                    .* :     *::  :: :       *:*  *     .  :  *    *

HcV-NS5A            HGARFFRATGG*D**RGSFHCR---RNPAAFQEVPSSSTNLG*ARLQSTLD*EVE 347
HGV-NS5A-2          MSSTWLFPY*KPCSHRAMPHAS*R*RCRAAWRRASRASFPWG*PWLTWLACAKWR 380
Score                .: :: .       *.  *.    *  **:...  :*   **. *      : .

HcV-NS5A            AAEL*PPGRSRLCFAT*KADPRPPP*EEADGRAFRVDG 385
HGV-NS5A-2          SRTIQPIVTRCALRLNCRLGAWWAMNLPLNVTSVR--- 415
Score               :  : *         . : ..  .     :  :.*
```
