# Supplementary material for: Whole-genome sequencing of human Pegivirus variant from an Egyptian patient co-infected with hepatitis C virus: a case report
Source: Virol J. 2019 Nov 11;16:132. doi: 10.1186/s12985-019-1242-5 (PMC6849219; doi:10.1186/s12985-019-1242-5)
Supplement: Supplementary file 5 — Additional file 5: Alignments of the NS5B protein structure of HCV and HPgV. [file 12985_2019_1242_MOESM5_ESM.pdf]

```
HGV-NS5B   --LGKRPWLLSPTSGLACRSRGPLRPSPLW*GRLARC*WLTPPRCMSPIQTTWGG
HcV-NS5b   PRLDCRLVVHRKRVGGCCVLLNVIFMD--WGAGNTLCG*GSQAANQPPEQLTAAP
Score        *. *  :     * .*   . :  .  * .  : *   : .   .* * * .

HGV-NS5B   EWTRLPSGVLPGFTTNSLWTLSSARERQLKPA*AWVTLMRRQ*GLLGHMLPWAGD
HcV-NS5b   S*HGVCHDLQVCHHTAEEGHLRPTPGGRQSLL*CPKGD*DPSVHREGSLAFCRGS
Score      .   :  .:   . * .   * .:   : .  *.       .    * :    *.

HGV-NS5B   LRCRSRTSPPLRGRWLSMTDFRRYLKGLRSHLP*L*KRRCSSKTVRRRRPPASLC
HcV-NS5b   LRPDAPPFC*IQIWLRGKGRSESF*QGR*PHQLRVGGLAGGPNHPHLNDHHGQK*
Score      **  : .   ::    .    . : :*  .*   :     ..:  : .   ..

HGV-NS5B   SLPWTSG*-LKS*SWGTRVEWPRRCWGGLTPSSTPQTSGLRRCSSCGNQRKPLAP
HcV-NS5b   GLLREAGEGRPKASASNRISRPRGPGLREESAL*RRPTTLHGHYGSFLRLPILSS
Score      .*   :*    . * ..*:. **       .:   :.: *:   ..  :   *:.

HGV-NS5B   SAWTRRASTVALLKRTWRWRR-SYMLWLQTTQSGCEPWGNTMLQAPWLPRKGFPS
HcV-NS5b   AAGRIPFVRVGFQEGSHGVLI*HPLL*LHCD*ERHQGRGGSLSVL*SGA*GSQGH
Score      :*       *.: : :        :* *:   .  :  *.::      .  .

HGV-NS5B   VRGTVDPQGF*PPARATA*PATLRCQPPAREWD*KMSRFSSRAMTV*SYANG---
HcV-NS5b   NRPHRETLRGRPHV*QQGRPMRQPQVPRERGFYHQLRKHTDVLP*GHSRHQGGRP
Score       *   :.    * .   . *      *  * :  :: :.:.      *  :*

HGV-NS5B   QCATRAKLWAEP*RAMGTRANLRIMHHWTRPPSAPLGLLSAMQMGSAISS*QRTF
HcV-NS5b   EGLHHAGLR**PGRHR*ERRC*GGQPSTSSFHGSYDKILGTPRRRPATSV*LGTD
Score      :   :* *   * *    *         :   .:   :*.: :  .* * *  *

HGV-NS5B   GGRSLACRASTVTLWPRPLVTSSFILGTPSHGGSSSRTC*RARFGVVAHRLILCG
HcV-NS5b   NILLLQCLSRTRCDRQKGLLPDPRP*NPPGSSSLGNSPTHSSQFLVG*HYTLRA-
Score      .   * * : *     : *:...   ..*. .. .. .   ::* *  *  : .
```

```
HGV-NS5B    ARYMVTTTSFHWTNCQTSSWPSTDQQR*GLPQTQPKQKWRPARC*--ATSSSRA*
HcV-NS5b    --YNLGAHGIDDPFLLNTPKPGSS*ESPRLRHVRSHILNHSAGSSSHNSETPRLE
Score         * : : .:. .   .:. *.:. :   * :.:.:   :.* .    :.:.*

HGV-NS5B    RSTVRRLEHCEPACFGRAVGPSWLGACCGIQAYGSPPPRLLVSRG-----VFPCP
HcV-NS5b    RVYAARILSARTQSGGRLPQETWGTPIESVETSGPSSPRQAHRPGREGQDLWPVP
Score       *  . *:  ... . **    :*  .  .::: *...**     *     ::* *

HGV-NS5B    PPTWG------------WFINWISQARGVAG-GGWGS*PC-
HcV-NS5b    L*LGGENQAQTHSIAGRCQARFIELVHGRRRRGGVGRASCR
Score           *               .:*. .:*    ** *  .*
```
